# Declining use of percutaneous coronary intervention across population groups, 2011-2022

**DOI:** 10.1093/haschl/qxag039

**Published:** 2026-03-10

**Authors:** Vadim M Shteyler, Jeffrey Tabas, Jessica N Holtzman, Yu-Chu Shen, Renee Y Hsia

**Affiliations:** Division of Pulmonary, Critical Care, Allergy, and Sleep Medicine, University of California San Francisco, San Francisco, CA 94143, United States; Philip R. Lee Institute for Health Policy Studies, University of California, San Francisco, CA 94143, United States; Department of Emergency Medicine, University of California, San Francisco, CA 94143, United States; Division of Cardiology, University of California San Francisco, San Francisco, CA 94143, United States; Department of Defense Management, Naval Postgraduate School, Monterey, CA 93943, United States; National Bureau of Economic Research, Cambridge, MA 02138, United States; Philip R. Lee Institute for Health Policy Studies, University of California, San Francisco, CA 94143, United States; Department of Emergency Medicine, University of California, San Francisco, CA 94143, United States

**Keywords:** percutaneous coronary intervention, health services, cardiology, socioeconomic status, utilization by race, cardiac catheterization

## Abstract

**Background:**

Coronary revascularization rates declined with decreasing acute coronary syndrome (ACS) and evolving appropriate use criteria (AUC). Population-based utilization trends by sociodemographic categories have not been assessed.

**Methods:**

We examined annual trends of all cardiac catheterizations and percutaneous coronary interventions (PCI) in California, 2011-2022, using the Health Care Access and Information database. We compared the PCI shares of cardiac catheterizations (“PCI shares”) between sociodemographic and facility-type strata. Regression models assessed significance of linear trends; pairwise comparisons assessed differences between strata.

**Results:**

We identified 1 839 444 cardiac catheterizations between 2011 and 2022. Catheterizations and PCI decreased from 2011 to 2013, plateaued, then decreased again in 2020. Higher median zip code family income, private insurance, and White or Asian/Pacific Islander, compared to Black or Hispanic, race were associated with higher PCI shares.

**Conclusions:**

Califoirnia's PCI share decreased, likely with decreasing ACS and AUC for coronary revascularization. Decreasing PCI but not cardiac catheterizations suggests that the contributors to fewer interventional procedures did not affect diagnostic procedure rates. Sociodemographic and payer differences in the PCI share persisted throughout the study, despite Medicaid expansion. The PCI share of cardiac catheterizations may be a useful measure of utilization heterogeneity across sociodemographic strata.

## Introduction

Over the past 2 decades, rates of acute coronary syndrome (ACS), hospital admissions for coronary artery disease (CAD), ST-elevation myocardial infarction (STEMI), and death after non-ST elevation myocardial infarction (NSTEMI) in the United States have all been shown to decline in large population-based studies.^1,2^ In parallel, observational studies showed declines in invasive cardiac procedural utilization as well as the proportion classified as inappropriate,^3-5^ correlating with improvements in preventative care, uptake of physiologically guided percutaneous coronary intervention (PCI), and increasing support for upfront medical rather than invasive therapies for stable CAD.^5-11^ The latter particularly shaped utilization with the release of American College of Cardiology and American Heart Association appropriate use criteria (AUC) for coronary revascularization in 2009 and widespread policies emphasizing their implementation over subsequent years.^12-15^ Recent years have also seen significant healthcare reform as the Affordable Care Act (ACA) expanded health insurance coverage and promoted substantial investments into reaching previously underserved communities, with both likely influencing cardiac procedure utilization. One study attributed a 2.9% decrease in cardiovascular mortality among middle-aged Medicaid beneficiaries to Medicaid expansion after ACA.^16^ In 2012 alone, California's non-profit hospitals invested about $4B toward previously unaddressed community health needs.^17^ Lastly, since March 2020, the COVID-19 pandemic profoundly influenced cardiac procedure utilization.^18^

Invasive cardiac procedure access and utilization, however, have varied widely by socioeconomic, demographic, and geographic factors.^19,20^ A large body of evidence shows lower PCI utilization among economically disadvantaged and racially minoritized patients across many contexts.^21-31^ Similarly, low-income and rural communities have been shown to have reduced access to PCI-capable centers and timely PCI.^32,33^ ACA adoption might have mitigated some of these differences.^34^ The subsequent COVID-19 pandemic, however, has had profoundly unequal effects, with the greatest increases in cardiovascular disease seen in racially minoritized groups.^35-37^ Together, many studies of large insurance and registry databases suggest that healthcare systems architecture, not just clinical indication, determines cardiac procedure utilization. However, insurance and registry data have several prominent limitations: they may include only participating institutions or enrollees from select insurers for as long as they remain enrolled, introducing selection bias and limiting generalizability. Often, they do not offer a complete population-based assessment of utilization trends across all insurers and hospital systems. Thus, though total rates of PCI have declined, population-level patterns of decline by racial identity, socioeconomic status, and community income level have not been assessed.

This analysis seeks to assess population-based trends of cardiac catheterizations, PCI, and the share of cardiac catheterizations that include PCI. We explore the latter as a population-based measure comparing differences in relative PCI utilization across social, racial, and economic categories, groups between which the ratio should not systematically differ. Systematic differences in PCI share of catheterizations across socioeconomic, racial, ethnic, or insurance groups would likely signal structural inequities operating through one or more pathways (eg, differential access to PCI-capable facilities, differences in baseline health at the time of catheterization, or varying severity of atherosclerotic disease at presentation) when the alternatives (ie, biological factors or patient preferences) are less plausible. As the epidemiologic landscape of atherosclerotic cardiovascular disease, appropriate use criteria for PCI, and healthcare access infrastructure all continue to evolve, population-wide assessments of invasive cardiac procedure utilization remain critical.

## Methods

### Data sources

This is a retrospective cohort analysis of non-public patient-level and healthcare facility-level longitudinal statewide data from the Department of Health Care Access and Information. We identified all patients who had undergone cardiac catheterization, percutaneous coronary intervention (PCI), coronary artery bypass graft (CABG) within 1 day of a cardiac catheterization, and the number of vessels revascularized with PCI in the state of California (CA) between January 1, 2011, and December 31, 2022, using Current Procedural Terminology (CPT) and International Classification of Disease (ICD) −9 and −10 Procedure codes (Appendix 1). This includes both inpatient PCI (eg, performed during a hospitalization episode) as well as outpatient PCI. Staged procedures are counted as separate PCI while same-day diagnostic and interventional cases are counted as a catheterization and PCI. We used discharge data to categorize the principal diagnosis for the procedure as “acute myocardial infarction,” “ischemic heart disease,” “angina,” or “other” and to determine if the primary encounter diagnosis was STEMI or not. We defined quantiles of median family income by the zip code of the patient's recorded mailing address. Teaching hospitals were defined by membership in the Council of American Teaching Hospitals. Publicly insured patients had Medicare, Medicaid, or indigent coverage.

Annual California population data were obtained from publicly available datasets from the U.S. Census Bureau website. Race population estimates were obtained from 2011 through 2022 from the U.S. Census Bureau and American Community Survey, sourced from USAFacts; missing 2015 data was imputed as the mean of 2014 and 2016 (see eMethods).

### Statistical analysis

We examined annual trends in total cardiac catheterization utilization rates (total annual procedures divided by the California population that year), PCI utilization rates, and the ratio of PCI to cardiac catheterizations (heretofore “PCI share of cardiac catheterizations” or “PCI share”) for all Californians between January 1, 2011, and December 31, 2022. We stratified these trends by sociodemographic (age, race, and socioeconomic status) and facility-specific (ownership, including for-profit, non-profit, and government; medical school affiliation; teaching hospital status) factors. The share of procedures paid by different insurance types was calculated by dividing the number of procedures paid by each insurer each year by the total California population that year.

We tested the significance of the observed annual temporal trends in cardiac catheterization rates and PCI rates with bivariate linear regression models and tested PCI shares of cardiac catheterizations with bivariate logistic models assessing odds of undergoing PCI conditional on having received cardiac catheterization (with a continuous calendar year variable as the predictor). Segmented regression models assessed for changing slopes of linear trends and located optimal changepoints. Model assumptions (linearity, normality of residuals, homoscedasticity, influential outliers) were tested. Multiple regression models assessed the statistical significance of stratification by each multilevel sociodemographic variable, in turn. We performed pairwise comparisons between the estimated marginal means of all levels of the sociodemographic variable. The *P*-values were adjusted for multiple comparisons using the Tukey method.

As a robustness analysis, we examined adjusted PCI shares of catheterization across race, payer, and income categories from a linear probability model (LPM) adjusted for age, gender, race, payer, and income, with year as a fixed-effects, and with cluster-robust standard errors. We compared these to adjusted PCI shares across race, payer, and income categories from an LPM with facility fixed-effects, to determine whether the observed disparities could be attributable to site differences.

All analyses were performed using R software version 4.3.1 for Windows. This study was not considered human subjects and therefore deemed exempt from UCSF Institutional Review Board review. The study followed the Strengthening the Reporting of Observational Studies in Epidemiology (STROBE) reporting guideline.

## Results

In this study of cardiac catheterization and PCI utilization in CA from January 1, 2011, to December 31, 2022, we observed 1 839 444 cardiac catheterizations, of which 561 969 (30.6%) included PCI. Female patients comprised 36.9% of cardiac catheterizations and 29.0% of PCI. Asian or Pacific Islander (Asian/PI), Black, Hispanic, and White patients comprised 9.9%, 6.6%, 21.9%, and 55.3% of cardiac catheterizations, and 10.8%, 5.1%, 19.9%, and 56.9% of the PCI (Table 1). Patients who had cardiac catheterizations had a mean age of 65.4 and those who had PCI had a mean age of 66.3. Non-profit, government, and University of California hospitals performed 72.4%, 5.4%, and 6.0% of cardiac catheterizations and 70.9%, 6.5%, and 4.2% of PCI (Table 1). Appendix 2 shows annual trends in these subgroup characteristics.

**Table 1.** Characteristics of cardiac catheterization patient encounters, with and without PCI, in California from 2011 to 2022.

	No PCI (*N* = 1 277 475)	PCI (*N* = 561 979)	All cardiac catheterizations (*N* = 1 839 454)
**Age** mean (SD)	65.0 (14.2)	66.3 (12.2)	65.4 (13.7)
**Female** mean (SD)	40.3% (49.1)	29.0% (45.4)	36.9% (48.2)
**Race**			
Asian/PI	120 691 (9.4%)	60 744 (10.8%)	181 435 (9.9%)
Black	92 056 (7.2%)	28 759 (5.1%)	120 815 (6.6%)
Hispanic	291 163 (22.8%)	112 088 (19.9%)	403 251 (21.9%)
White	696 763 (54.5%)	319 530 (56.9%)	1 016 293 (55.2%)
Additional category	76 802 (6.0%)	40 858 (7.3%)	117 660 (6.4%)
**Insurance**			
Medicare	702 889 (55.0%)	300 320 (53.4%)	1 003 209 (54.5%)
Medicaid	183 800 (14.4%)	72 940 (13.0%)	256 740 (14.0%)
Private	342 395 (26.8%)	164 775 (29.3%)	507 170 (27.6%)
Self-pay	17 998 (1.4%)	11 578 (2.1%)	29 576 (1.6%)
Other	30 393 (2.4%)	12 366 (2.2%)	42 759 (2.3%)
**Income** ^a^			
Lowest income	366 063 (28.7%)	142 318 (25.3%)	508 381 (27.6%)
Medium Income	367 407 (28.8%)	155 144 (27.6%)	522 551 (28.4%)
Highest income	523 953 (41.0%)	255 926 (45.5%)	779 879 (42.4%)
Missing	20 052 (1.6%)	8591 (1.5%)	28 643 (1.6%)
**Principal diagnosis category**			
Acute MI	169 089 (13.2%)	257 899 (45.9%)	426 988 (23.2%)
Angina	11 113 (0.9%)	1550 (0.3%)	12 663 (0.7%)
Ischemic heart disease	405 526 (31.7%)	229 769 (40.9%)	635 295 (34.5%)
Other	691 747 (54.1%)	72 761 (12.9%)	764 508 (41.6%)
**Facility ownership**			
Government	80 254 (6.3%)	36 432 (6.5%)	116 686 (6.3%)
Investor	182 610 (14.3%)	84 326 (15.0%)	266 936 (14.5%)
Non-profit	897 712 (70.3%)	407 023 (72.4%)	1 304 735 (70.9%)
University of California	86 044 (6.7%)	23 613 (4.2%)	109 657 (6.0%)
Missing	30 855 (2.4%)	10 585 (1.9%)	41 440 (2.3%)
**Medical school-affiliated** ^b^	58.7% (49.2)	56.0% (49.6)	57.9% (49.4)
Missing	83 977 (6.6%)	27 226 (4.8%)	111 203 (6.0%)
**Teaching hospital** ^c^	315 331 (24.7%)	116 874 (20.8%)	432 205 (23.5%)

Abbreviations: MI, Myocardial infarction; PCI, Percutaneous Coronary Intervention; SD, Standard deviation.

^a^Tertiles of median family income by zip code.

^b^Residency program with medical school affiliation.

^c^Members of the Council of American Teaching Hospitals.

**Figure 1.**
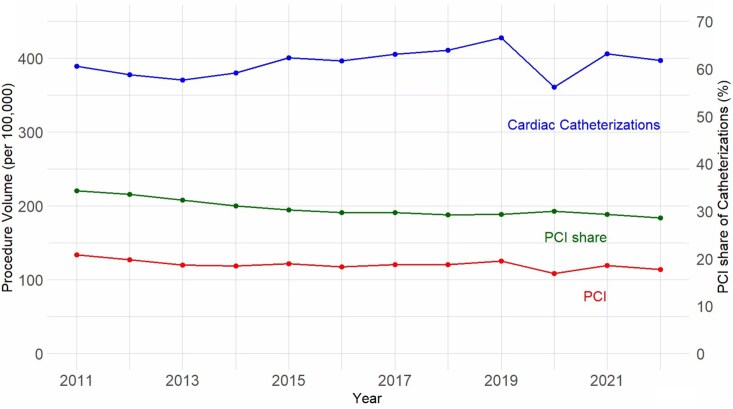
Trends in annual cardiac catheterization and PCI utilization rates and in the proportion of cardiac catheterizations including PCI, 2011-2022. PCI: Percutaneous Coronary Intervention.

**Figure 2.**
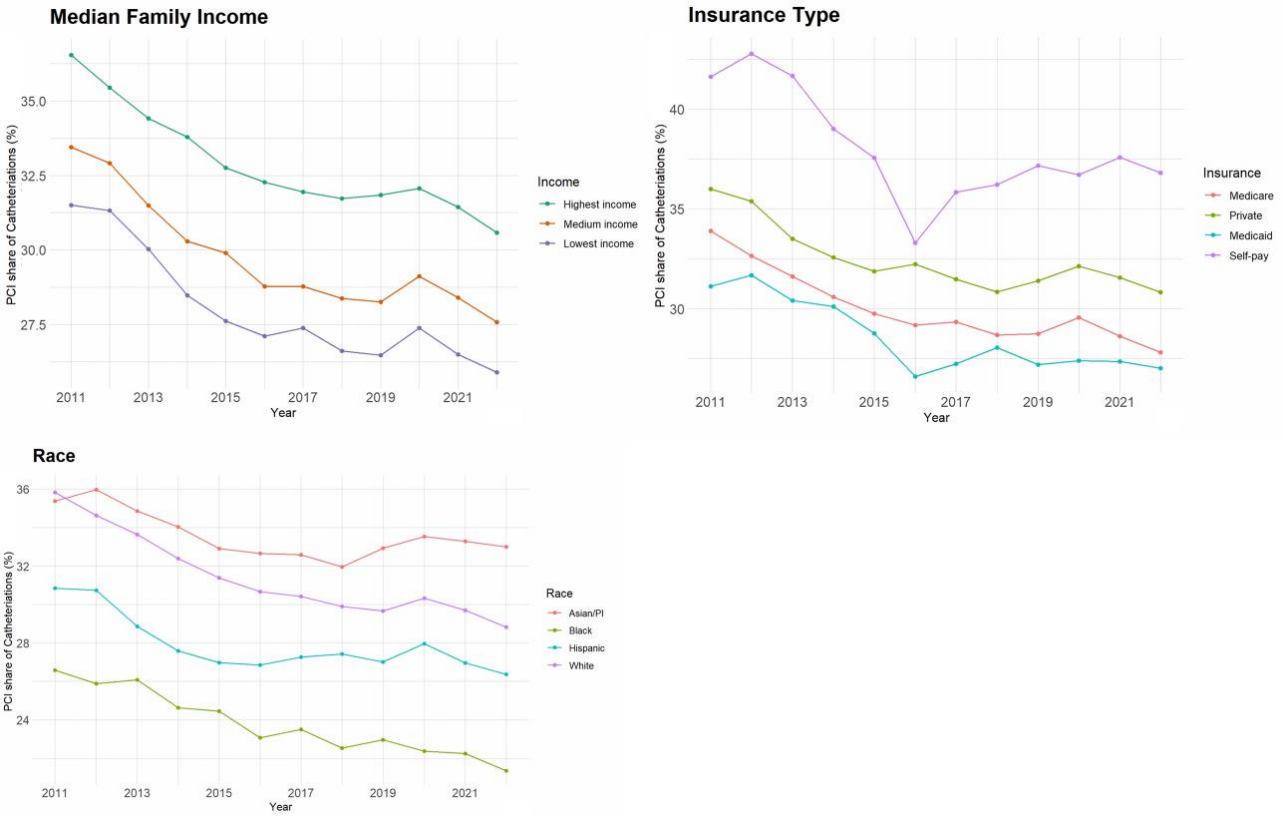
Proportions of cardiac catheterizations involving PCI by median zip code household income, insurance type, and race categories, 2011-2022. PCI: Percutaneous Coronary Intervention.

Trends in rates of cardiac catheterization and PCI are shown in Figure 1. Rates of both procedures dropped more from 2011 to 2013 than in subsequent years, which was significant for PCI rates in a segmented regression model (*P* = 0.035). The decrease was mostly observed among White patients, among residents of high-income neighborhoods, and for an ischemic heart disease indication (Figure 2). There was another observed relative decrease in rates of both procedures in 2020, associated with the start of the COVID-19 pandemic, which largely rebounded by 2022 but did not return to 2019 rates. The decrease was seen in all categories of family income, gender, race, insurance coverage, and for facilities whether they were for-profit, government-owned, medical school-affiliated or not. The decreased utilization persisted for STEMI, acute myocardial infarctions, and ischemic heart disease diagnoses. Both in absolute numbers and rates per 100,000, White patients had the highest rates of cardiac catheterization and PCI, while Hispanic patients had the lowest utilization rates (Appendix 3). The rate of cardiac catheterizations and PCI (per 100 000) paid by Medicaid increased most compared to other insurance types, from 36.5 to 54.9 catheterizations (50.4% increase) and from 10.2 to 16.4 PCI (61.0% increase) from 2013 to 2014, with near elimination of procedures paid by indigent care and a modest decrease in self-pay during this time (Appendix 4).

The PCI share of cardiac catheterizations decreased over the course of the observation period 2011-2022, save for a transient increase in 2020 (Figure 1); the linear decrease was statistically significant in a bivariate logistic model (*P* < 0.001). This proportion was decreasing across nearly all sociodemographic and facility variables. We found patients living in the highest income areas to have the highest PCI shares of cardiac catheterizations. The PCI share of cardiac catheterizations was highest for Asian/PI patients, followed by White, Hispanic, and Black patients. Self-paying patients had the highest PCI shares, followed by those with private, Medicare, and then Medicaid/Indigent coverage (Figure 2). These trends persisted after stratification by various facility-level categories. All of the strata in each of the variables in Figure 2 were statistically significantly different in pairwise comparisons of logistic models (*P* < 0.001 for each pair). The race, payer, and income category differences persisted in a LPM controlling for age, gender, race, payer, and income categories (Appendix 5).

**Figure 3.**
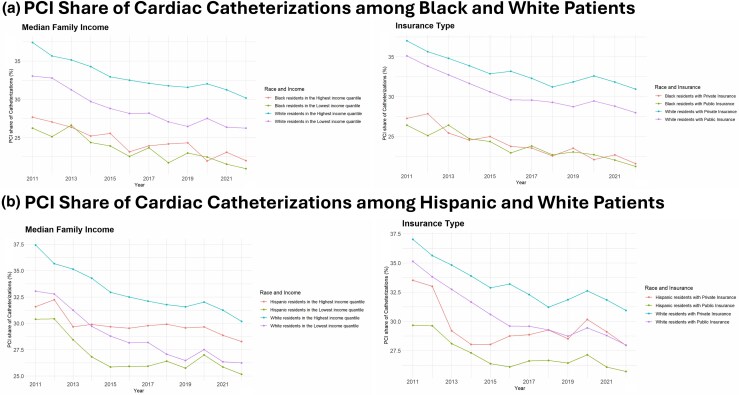
Proportions of cardiac catheterizations involving PCI among patients at the extremes of median zip code household income and state ADI scores and privately vs publicly insured identifying with (A) Black vs White and (B) Hispanic vs White race categories, 2011-2022. PCI: Percutaneous Coronary Intervention.

Comparing PCI shares between race categories and the extremes of income or insurance categories reveals significant sorting. White and Hispanic patients had sorted along socioeconomic divides, with differences in the PCI share of cardiac catheterizations being much wider between high and low income and private or public insurance types than between the racial categories. However, the PCI shares of cardiac catheterizations between Hispanic and White patients of the same socioeconomic strata have been converging over time. Conversely, regardless of income or insurance coverage provider, Black patients had consistently lower PCI shares of cardiac catheterization than other groups (Figure 3). This disparity persisted across facility types (Appendix 6).

Sensitivity analyses showed similar disparities when restricted by diagnosis group (acute myocardial infarction, AMI; ischemic heart disease, IHD; STEMI), when analyzing revascularization with either PCI or coronary artery bypass graft (CABG) within one day (as competing variables), and when analyzing the total number of revascularized vessels rather than the number of procedures (Appendix 7). Adjusted differences in PCI shares across race and payer categories persisted in a LPM with facility fixed effects and cluster-robust standard errors, whereas differences across income categories were fully attenuated, suggesting that income-based differences may reflect between-facility variation like from different facility-level practice patterns or differential sorting of patients across facilities (Appendix 8).

## Discussion

This large population analysis of all cardiac catheterizations and PCI performed in CA from 2011 through 2022 reveals several important macro-level utilization trends. Though we cannot causally attribute the observed utilization trends to any one factor—evolving AUC, healthcare reforms, or a global pandemic—viewing these trends as a moving equilibrium between them offers insights. We note a statistically significant decrease in state-wide PCI utilization from 2011 to 2013 that we hypothesize to be potentially attributable to multiple society guidelines and subsequent quality assurance measures emphasizing AUC between 2009 and 2012.^13-15^ Descriptively, this early decrease was greatest for patients in the White race category and top quantiles of economic advantage, consistent with our expectations about the populations in which efforts to curb overutilization might have the greatest impact. We did not see concomitant decreases in PCI utilization among Medicaid beneficiaries or the lowest income quantile.

In 2014-2019, we observed a relative plateau in PCI utilization despite additional factors expected to further decrease PCI utilization—increasing evidence supporting upfront medical management of stable coronary disease,^38^ physiologically-guided revascularization,^39^ and growing value-based payment models.^40^ This may reflect a counterbalancing effect of insurance expansion associated with the ACA implementation occurring during that period. Broadly, the decreasing PCI rates, the stable or increasing cardiac catheterization rates, and the consequent statistically significant decreasing PCI share of cardiac catheterizations suggest that if performance guidelines contributed to fewer patients selected for PCI, they may not have similarly impacted decisions about diagnostic evaluation.

Many studies demonstrate improved access and outcomes after ACA implementation.^41-43^ Our study shows additional trends that temporally correlate and may be consistent with ACA implementation. Starting in 2014, we observed sustained increases in cardiac catheterization and PCI rates among insured individuals, with the greatest increase seen among Medicaid beneficiaries. Further, the lowest income quantile saw the highest increase in utilization of both procedures. Both observations may suggest improved access related to known expanded coverage with ACA implementation at that time.

We observed meaningful and persisting disparities in relative PCI utilization as a share of cardiac catheterizations among socioeconomically disadvantaged and racially minoritized patients. Living in an economically advantaged neighborhood with higher median family income, having private insurance, or identifying as White or Asian/PI (compared to Black or Hispanic) was each associated with a statistically significant higher relative PCI share, independent of overall secular trends. The uniform separation of PCI shares of catheterization observed across quantiles of the sociodemographic measures, persisting over time, makes differences in clinical need a less plausible explanation. Indeed, increased rates of cardiovascular disease in economically disadvantaged and racially minoritized groups would be expected to increase PCI shares in those strata, a pattern that would counter rather than explain our finding. In subgroup analyses, even the potential benefits of ACA expansion may not have been equally distributed. The increased PCI rates among Medicaid beneficiaries during ACA expansion were largely driven by increases among White patients, suggesting that insurance coverage might not be the only access barrier in other groups.

Our findings are consistent with prior studies showing lower PCI utilization among economically disadvantaged and racially minoritized patients in many contexts.^21-30^ One study examining acute myocardial infarctions in New York State in 1995 found a similar gradient in the odds of revascularization across quintiles of income.^31^ Disappointingly, our study shows that these disparities remain decades later and persist from 2011 to 2022, despite the significant healthcare reforms, ACA adoption, and investments in health equity during this interval.^44^

Our results showing persistent disparities across racial and socioeconomic categories contain an important caveat. Over the duration of our observation period, we observed shifts in racial sorting in the PCI share of cardiac catheterizations among the privately vs publicly insured and the extreme quantiles of income. We found that PCI shares were similar among Hispanic patients across the socioeconomic spectrum and different from White patients in the earlier years of our observation period. However, over time, we noted a shift toward greater sorting along socioeconomic categories, with economically advantaged Hispanic patients having PCI shares increasingly similar to economically advantaged White patients rather than economically disadvantaged Hispanic communities. Despite this “re-sorting,” the overall socioeconomic disparities persisted. The reason for the re-sorting is unclear, but the observation reinforces that race is not an immutable determinant of utilization but rather a social construct that interacts dynamically with socioeconomic forces. Black communities, conversely, had very similar PCI shares regardless of their insurance coverage or neighborhood income throughout the study, which were lower than those of White patients from any category. This is analogous to a prior study finding that higher income was associated with improved outcomes after PCI for White, but not Black, patients.^45^ Unlike a prior study that found that decreased utilization of nonavoidable Emergency Department visits during the COVID-19 pandemic only persisted though 2022 among socially disadvantaged groups,^46^ we found decreased PCI utilization persisting through 2022 across socioeconomic strata. The transient increase in the PCI share in 2020 may reflect more judicious cardiac catheterizations that identify intervenable coronary lesions with more specificity and a shift toward later-presenting disease.

Several factors may plausibly cause disparate PCI shares of cardiac catheterizations. Patients with decreased access to subspeciality interventional cardiology care more likely undergo separate diagnostic and therapeutic procedures, lowering their PCI share and increasing the costs and risks of treatment. Economically disadvantaged or racially minoritized patients may present with more advanced disease or at lower levels of baseline health, reflecting disparate access to and effectiveness of upstream health resources that may make them worse PCI candidate. Differentially prevalent coronary disease mimics (eg, stress cardiomyopathy), increasing catheterization use without PCI, may reflect disparities in those contexts. Implicit bias or interpersonal racism may also contribute. As the alternatives to disparities, biological determinants or patient preferences for catheterization but not revascularization, would less plausibly align so consistently with social hierarchies, they are less plausibly explanatory.

Importantly, our results may reflect overutilization in advantaged sociodemographic strata. Either through increased risk-aversion, economic incentives, or litigation concerns, clinician decisions may contribute to higher PCI shares among advantaged patients, disparately increasing the risks and costs of treatment.^44^

Many of the observed trends in the PCI share of cardiac catheterizations are, unfortunately, unsurprising. Their consistency with prior findings and alignment with expectations about the effects of policies, however, lend the ratio credibility as a promising population-level candidate measure highlighting potentially unwarranted variability in PCI utilization between sociodemographic groups. PCI share of cardiac catheterizations should not differ by socioeconomic or racial category.

It is critical to understand that the crude PCI share of cardiac catheterizations does not contain the relevant patient-level clinical information to assess utilization appropriateness, thereby limiting its usefulness as an overall healthcare metric. Yet it may be a useful population-wide surveillance measure of disparities in invasive revascularization, in part, because it is not a reported quality metric that would be targeted or tracked. Such trends in PCI shares may help generate hypotheses about or contextualize individual-level analyses and policy priorities. Further studies linking subgroup differences in PCI shares to outcomes such as mortality or readmission would further the clinical significance of these utilization patterns. While population-based evidence is appropriate for surveillance, informing targeted countermeasures requires patient-level adjudication of procedural overuse or underuse and determining the mechanisms behind our observed disparities.

## Limitations

This study has important limitations. First, this is a population-based study without access to granular, patient-level clinical variables and therefore cannot adjudicate the appropriateness of any single intervention. Instead, we offer evidence of predictable procedure utilization differences across socioeconomic and demographic categories. Next, while we stratify our analyses by multiple levels of race and socioeconomic status, those strata may contain heterogeneity. For example, income distributions for Black and White patients may differ within the same quintile of median family income. We balance our goal for homogeneous strata with our goal for maintaining adequate sample sizes for each stratum in each year to elucidate important trends, accepting some imprecisions. Similarly, our strata may misclassify. The economic disadvantage of Black, urban patients, for example, may be underestimated by high-cost city living and neighboring wealthy communities. This may increase apparent racial sorting by economic advantage but not the overall observed racial disparities. The transition from ICD-9 to ICD-10 may introduce comparison errors due to imperfect code mapping but should not affect comparisons between groups in a given year or comparisons in years before 2015 and after 2016. In keeping with prior population-based studies, our definition of cardiac catheterizations includes right-heart catheterizations.^47-49^ This unlikely influences the conclusions as they likely represent a small fraction of total procedures. The catheterizations are defined consistently over the study interval, so there would need to be a significant systematic change in utilization of those other catheterizations to affect our observed trends. We observe similar trends in our indication-specific sensitivity analyses, which likely exclude those other catheterizations, suggesting the robustness of our results to more narrowly defined denominators.

Lastly, though the analysis comprises over 11% of the U.S. population and about 9.8% of U.S. cardiologists, the results may not generalize to other States.^50^ California law requires a higher level of care for PCI facilities than most States and requires utilization and outcomes reporting; this may influence State-wide PCI utilization throughout the duration of the study.^51^ As the factors plausibly increasing PCI use (ACA implementation) and decreasing PCI use (AUC enforcement) over the study period varied by State, the timing and magnitude of the observed trends may differ between jurisdictions.^52^ These major suspected influencers of PCI utilization trends in this study, however, were national policies or guidelines and the COVID-19 pandemic, so we would expect similar trends nationwide.

## Conclusion

This large population study of cardiac catheterization and PCI utilization rates from 2011 through 2022 found decreasing PCI but not cardiac catheterization rates preceding the COVID-19 pandemic, suggesting that AUC may have reduced the number of patients selected for treatment but not diagnostic procedures. Our study observations are consistent with increased cardiac procedure utilization after ACA implementation, corroborating the role for insurance expansion in increased access. Our subgroup analyses, however, also suggest that its benefits may have distributed unequally and that socioeconomically disadvantaged, publicly insured, and racially minoritized patients were consistently less likely to undergo PCI after cardiac catheterization. The disparities persisted throughout the study despite the significant healthcare reforms of that period, including ACA implementation.

## Supplementary Material

qxag039_Supplementary_Data
