# Supplementary material for: Declining use of percutaneous coronary intervention across population groups, 2011-2022
Source: Health Aff Sch. 2026 Mar 10;4(3):qxag039. doi: 10.1093/haschl/qxag039 (PMC12974569; doi:10.1093/haschl/qxag039)
Supplement: qxag039_Supplementary_Data [file qxag039_supplementary_data.zip › Supplement_Declining PCI_Revised.docx]

**Declining Use of Percutaneous Coronary Intervention Across Population Groups, 2011-2022**

**Supplemental Document.**

**eMethods**

Annual California population data were obtained from publicly available datasets from the U.S. Census Bureau website: <https://www.census.gov/programs-surveys/popest/data/data-sets.All.List_221933341.html#list-tab-List_221933341>

Synthesized U.S. Census Bureau and American Community Survey data from USAFacts: <https://usafacts.org/data/topics/people-society/population-and-demographics/our-changing-population/state/california/>

| **Cardiac Catheterizations** | | | |
| --- | --- | --- | --- |
| **ICD-9** | **ICD-10** | **CPT** | **HCPCS** |
| 3721 | 4A020N6 | 92978 | G0269 |
| 3722 | 4A020N7 | 92979 |  |
| 3723 | 4A020N8 | 93451 |  |
| 8850 | 4A023N6 | 93452 |  |
| 8852 | 4A023N7 | 93453 |  |
| 8853 | 4A023N8 | 93454 |  |
| 8854 | 4A027N6 | 93455 |  |
| 8855 | 4A027N7 | 93456 |  |
| 8856 | 4A027N8 | 93457 |  |
| 8857 | 4A028N6 | 93458 |  |
|  | 4A028N7 | 93459 |  |
|  | 4A028N8 | 93460 |  |
|  | B200xZZ | 93461 |  |
|  | B201xZZ | 93462 |  |
|  | B202xZZ | 93463 |  |
|  | B203xZZ | 93464 |  |
|  | B204xZZ | 93505 |  |
|  | B205xZZ | 93563 |  |
|  | B206xZZ | 93564 |  |
|  | B207xZZ | 93565 |  |
|  | B208xZZ | 93566 |  |
|  | B20FxZZ | 93567 |  |
|  | B210xZZ | 93568 |  |
|  | B211xZZ | 93571 |  |
|  | B212xZZ | 93572 |  |
|  | B213xZZ | 93593 |  |
|  | B214xZZ | 93594 |  |
|  | B215xZZ | 93595 |  |
|  | B216xZZ | 93596 |  |
|  | B217xZZ | 93597 |  |
|  | B218xZZ | 93598 |  |
|  | B21FxZZ |  |  |

**Appendix 1.** PCI and Cardiac Catheterization Procedure Codes

|  | | | |
| --- | --- | --- | --- |
| **PCI** | | | |
| **ICD-9** | **ICD-10** | **CPT** | **HCPCS** |
| 0066 | 02703xx | 92920 | C9600 |
| 3601 | 02704xx | 92921 | C9601 |
| 3602 | 02713xx | 92924 | C9602 |
| 3605 | 02714xx | 92925 | C9603 |
| 3606 | 02723xx | 92928 | C9604 |
| 3607 | 02724xx | 92929 | C9605 |
|  | 02733xx | 92933 | C9606 |
|  | 02734xx | 92934 | C9607 |
|  | 02C03Z6 | 92937 | C9608 |
|  | 02C03ZZ | 92938 |  |
|  | 02C04Z6 | 92941 |  |
|  | 02C04ZZ | 92943 |  |
|  | 02C13Z6 | 92944 |  |
|  | 02C13ZZ | 92973 |  |
|  | 02C14Z6 | 92975 |  |
|  | 02C14ZZ | 92978 |  |
|  | 02C23Z6 | 92979 |  |
|  | 02C23ZZ | 93571 |  |
|  | 02C24Z6 | 93572 |  |
|  | 02C24ZZ | 92980 |  |
|  | 02C33Z6 | 92981 |  |
|  | 02C33ZZ | 92982 |  |
|  | 02C34Z6 | 92984 |  |
|  | 02C34ZZ | 92995 |  |
|  | 02H03DZ | 92996 |  |
|  | 02H03YZ |  |  |
|  | 02H13DZ |  |  |
|  | 02H13YZ |  |  |
|  | 02H23DZ |  |  |
|  | 02H23YZ |  |  |
|  | 02H33DZ |  |  |
|  | 02H33YZ |  |  |
|  | X2C0361 |  |  |
|  | X2C1361 |  |  |
|  | X2C2361 |  |  |
|  | X2C3361 |  |  |

**Appendix 2.** Characteristics of cardiac catheterization patient encounters, with and without PCI, in California from 2011 to 2022

|  | **2011** | | **2012** | | **2013** | | **2014** | | | **2015** | | **2016** | |  |
| --- | --- | --- | --- | --- | --- | --- | --- | --- | --- | --- | --- | --- | --- | --- |
|  | **N**o **P**CI **(N=96317)** | **P**CI **(N=50288)** | **N**o **P**CI **(N=95426)** | **P**CI **(N=48153)** | **N**o **P**CI **(N=96032)** | **P**CI **(N=45820)** | | **N**o **P**CI **(N=101117)** | **P**CI **(N=45769)** | **N**o **P**CI **(N=108908)** | **P**CI **(N=47373)** | **N**o **P**CI **(N=109412)** | **P**CI **(N=46167)** | |
| **Age** Mean (SD) | 64.0 (14.5) | 65.8 (12.4) | 64.2 (14.4) | 65.6 (12.5) | 64.4 (14.4) | 65.8 (12.5) | | 64.5 (14.3) | 65.9 (12.4) | 64.5 (14.2) | 65.9 (12.2) | 64.7 (14.2) | 66.0 (12.1) | |
| **Female** Mean (SD) | 0.417 (0.493) | 0.307 (0.461) | 0.415 (0.493) | 0.300 (0.458) | 0.403 (0.491) | 0.299 (0.458) | | 0.403 (0.491) | 0.296 (0.457) | 0.404 (0.491) | 0.295 (0.456) | 0.406 (0.491) | 0.289 (0.453) | |
| **Race** |  |  |  |  |  |  | |  |  |  |  |  |  | |
| Additional category | 5091 (5.3%) | 3146 (6.3%) | 4944 (5.2%) | 3012 (6.3%) | 5060 (5.3%) | 2759 (6.0%) | | 5558 (5.5%) | 3026 (6.6%) | 5992 (5.5%) | 3220 (6.8%) | 6381 (5.8%) | 3165 (6.9%) | |
| Asian/PI | 8388 (8.7%) | 4591 (9.1%) | 8084 (8.5%) | 4541 (9.4%) | 8460 (8.8%) | 4528 (9.9%) | | 9244 (9.1%) | 4768 (10.4%) | 10297 (9.5%) | 5049 (10.7%) | 10355 (9.5%) | 5020 (10.9%) | |
| Black | 7384 (7.7%) | 2674 (5.3%) | 7201 (7.5%) | 2515 (5.2%) | 7234 (7.5%) | 2552 (5.6%) | | 7443 (7.4%) | 2433 (5.3%) | 8013 (7.4%) | 2594 (5.5%) | 7894 (7.2%) | 2366 (5.1%) | |
| Hispanic | 19959 (20.7%) | 8903 (17.7%) | 20310 (21.3%) | 9014 (18.7%) | 21542 (22.4%) | 8738 (19.1%) | | 22866 (22.6%) | 8708 (19.0%) | 24729 (22.7%) | 9132 (19.3%) | 25218 (23.0%) | 9260 (20.1%) | |
| White | 55495 (57.6%) | 30974 (61.6%) | 54887 (57.5%) | 29071 (60.4%) | 53736 (56.0%) | 27243 (59.5%) | | 56006 (55.4%) | 26834 (58.6%) | 59877 (55.0%) | 27378 (57.8%) | 59564 (54.4%) | 26356 (57.1%) | |
| **Insurance** |  |  |  |  |  |  | |  |  |  |  |  |  | |
| Medicare | 50813 (52.8%) | 26059 (51.8%) | 50970 (53.4%) | 24711 (51.3%) | 51672 (53.8%) | 23893 (52.1%) | | 54006 (53.4%) | 23803 (52.0%) | 58346 (53.6%) | 24712 (52.2%) | 59295 (54.2%) | 24442 (52.9%) | |
| Private | 28262 (29.3%) | 15898 (31.6%) | 27400 (28.7%) | 14998 (31.1%) | 26943 (28.1%) | 13570 (29.6%) | | 28098 (27.8%) | 13570 (29.6%) | 30005 (27.6%) | 14044 (29.6%) | 29052 (26.6%) | 13813 (29.9%) | |
| Medicaid | 11786 (12.2%) | 5326 (10.6%) | 11566 (12.1%) | 5362 (11.1%) | 12136 (12.6%) | 5302 (11.6%) | | 15017 (14.9%) | 6471 (14.1%) | 16922 (15.5%) | 6837 (14.4%) | 17601 (16.1%) | 6380 (13.8%) | |
| Self-pay | 2657 (2.8%) | 1894 (3.8%) | 2615 (2.7%) | 1954 (4.1%) | 2470 (2.6%) | 1764 (3.8%) | | 1424 (1.4%) | 911 (2.0%) | 1209 (1.1%) | 727 (1.5%) | 1152 (1.1%) | 575 (1.2%) | |
| Other | 2799 (2.9%) | 1111 (2.2%) | 2875 (3.0%) | 1128 (2.3%) | 2811 (2.9%) | 1291 (2.8%) | | 2572 (2.5%) | 1014 (2.2%) | 2426 (2.2%) | 1053 (2.2%) | 2312 (2.1%) | 957 (2.1%) | |
| **Income** |  |  |  |  |  |  | |  |  |  |  |  |  | |
| Highest income | 40469 (42.0%) | 23302 (46.3%) | 38629 (40.5%) | 21211 (44.0%) | 38675 (40.3%) | 20298 (44.3%) | | 39426 (39.0%) | 20116 (44.0%) | 42473 (39.0%) | 20685 (43.7%) | 42156 (38.5%) | 20087 (43.5%) | |
| Medium income | 27769 (28.8%) | 13955 (27.8%) | 27492 (28.8%) | 13485 (28.0%) | 27057 (28.2%) | 12436 (27.1%) | | 28564 (28.2%) | 12414 (27.1%) | 29513 (27.1%) | 12590 (26.6%) | 30853 (28.2%) | 12464 (27.0%) | |
| Lowest income | 26494 (27.5%) | 12189 (24.2%) | 27892 (29.2%) | 12717 (26.4%) | 28817 (30.0%) | 12367 (27.0%) | | 31576 (31.2%) | 12569 (27.5%) | 35120 (32.2%) | 13397 (28.3%) | 34633 (31.7%) | 12877 (27.9%) | |
| **Principal diagnosis category** |  |  |  |  |  |  | |  |  |  |  |  |  | |
| Acute MI | 11803 (12.3%) | 21752 (43.3%) | 12168 (12.8%) | 22146 (46.0%) | 12513 (13.0%) | 22143 (48.3%) | | 12802 (12.7%) | 22197 (48.5%) | 14450 (13.3%) | 22752 (48.0%) | 15258 (13.9%) | 21419 (46.4%) | |
| Angina | 1035 (1.1%) | 161 (0.3%) | 996 (1.0%) | 187 (0.4%) | 1021 (1.1%) | 185 (0.4%) | | 1152 (1.1%) | 233 (0.5%) | 1142 (1.0%) | 264 (0.6%) | 840 (0.8%) | 85 (0.2%) | |
| Ischemic Heart Disease | 32034 (33.3%) | 23194 (46.1%) | 30340 (31.8%) | 20811 (43.2%) | 31608 (32.9%) | 18432 (40.2%) | | 33568 (33.2%) | 18052 (39.4%) | 34613 (31.8%) | 18557 (39.2%) | 35232 (32.2%) | 18980 (41.1%) | |
| Other | 51445 (53.4%) | 5181 (10.3%) | 51922 (54.4%) | 5009 (10.4%) | 50890 (53.0%) | 5060 (11.0%) | | 53595 (53.0%) | 5287 (11.6%) | 58703 (53.9%) | 5800 (12.2%) | 58082 (53.1%) | 5683 (12.3%) | |
| **Facility Ownership** |  |  |  |  |  |  | |  |  |  |  |  |  | |
| Government | 7163 (7.4%) | 3419 (6.8%) | 6405 (6.7%) | 3427 (7.1%) | 6520 (6.8%) | 3421 (7.5%) | | 6336 (6.3%) | 3394 (7.4%) | 6869 (6.3%) | 3478 (7.3%) | 6735 (6.2%) | 3082 (6.7%) | |
| Investor | 13175 (13.7%) | 7184 (14.3%) | 13224 (13.9%) | 6840 (14.2%) | 12639 (13.2%) | 6777 (14.8%) | | 14199 (14.0%) | 7168 (15.7%) | 16168 (14.8%) | 7655 (16.2%) | 17136 (15.7%) | 7762 (16.8%) | |
| Missing | 1728 (1.8%) | 770 (1.5%) | 1598 (1.7%) | 730 (1.5%) | 1867 (1.9%) | 600 (1.3%) | | 2264 (2.2%) | 598 (1.3%) | 2516 (2.3%) | 641 (1.4%) | 2489 (2.3%) | 628 (1.4%) | |
| Non-Profit | 68432 (71.0%) | 37130 (73.8%) | 68456 (71.7%) | 35239 (73.2%) | 69320 (72.2%) | 33223 (72.5%) | | 71829 (71.0%) | 32731 (71.5%) | 76490 (70.2%) | 33719 (71.2%) | 75533 (69.0%) | 32871 (71.2%) | |
| University of California | 5819 (6.0%) | 1785 (3.5%) | 5743 (6.0%) | 1917 (4.0%) | 5686 (5.9%) | 1799 (3.9%) | | 6489 (6.4%) | 1878 (4.1%) | 6865 (6.3%) | 1880 (4.0%) | 7519 (6.9%) | 1824 (4.0%) | |

|  | **2017** | | **2018** | | | **2019** | | **2020** | | **2021** | | | **2022** | |
| --- | --- | --- | --- | --- | --- | --- | --- | --- | --- | --- | --- | --- | --- | --- |
|  | **No PCI (N=112503)** | **PCI (N=47482)** | **No PCI (N=114862)** | **PCI (N=47549)** | **No PCI (N=119577)** | | **PCI (N=49558)** | **No PCI (N=99949)** | **PCI (N=42771)** | **No PCI (N=112341)** | **PCI (N=46597)** | **No PCI (N=111031)** | | **PCI (N=44452)** |
| **Age** Mean (SD) | 65.0 (14.1) | 66.4 (12.1) | 65.1 (14.1) | 66.5 (12.1) | 65.6 (14.0) | | 66.8 (12.0) | 65.4 (14.3) | 66.8 (12.0) | 65.8 (14.1) | 67.1 (12.0) | 66.2 (13.9) | | 67.3 (11.9) |
| **Female** Mean (SD) | 0.403 (0.491) | 0.286 (0.452) | 0.401 (0.490) | 0.287 (0.452) | 0.398 (0.489) | | 0.284 (0.451) | 0.396 (0.489) | 0.281 (0.449) | 0.398 (0.490) | 0.280 (0.449) | 0.398 (0.490) | | 0.280 (0.449) |
| **Race** |  |  |  |  |  | |  |  |  |  |  |  | |  |
| Additional category | 6611 (5.9%) | 3351 (7.1%) | 6832 (5.9%) | 3429 (7.2%) | 7846 (6.6%) | | 4095 (8.3%) | 6876 (6.9%) | 3731 (8.7%) | 7788 (6.9%) | 4043 (8.7%) | 7823 (7.0%) | | 3881 (8.7%) |
| Asian/PI | 10902 (9.7%) | 5271 (11.1%) | 11269 (9.8%) | 5291 (11.1%) | 11559 (9.7%) | | 5674 (11.4%) | 9242 (9.2%) | 4663 (10.9%) | 11402 (10.1%) | 5690 (12.2%) | 11489 (10.3%) | | 5658 (12.7%) |
| Black | 7879 (7.0%) | 2420 (5.1%) | 8307 (7.2%) | 2417 (5.1%) | 8422 (7.0%) | | 2510 (5.1%) | 6982 (7.0%) | 2012 (4.7%) | 7871 (7.0%) | 2251 (4.8%) | 7426 (6.7%) | | 2015 (4.5%) |
| Hispanic | 26185 (23.3%) | 9810 (20.7%) | 26920 (23.4%) | 10170 (21.4%) | 27609 (23.1%) | | 10220 (20.6%) | 22936 (22.9%) | 8904 (20.8%) | 26348 (23.5%) | 9725 (20.9%) | 26541 (23.9%) | | 9504 (21.4%) |
| White | 60926 (54.2%) | 26630 (56.1%) | 61534 (53.6%) | 26242 (55.2%) | 64141 (53.6%) | | 27059 (54.6%) | 53913 (53.9%) | 23461 (54.9%) | 58932 (52.5%) | 24888 (53.4%) | 57752 (52.0%) | | 23394 (52.6%) |
| **Insurance** |  |  |  |  |  | |  |  |  |  |  |  | |  |
| Medicare | 61402 (54.6%) | 25505 (53.7%) | 63388 (55.2%) | 25500 (53.6%) | 67692 (56.6%) | | 27304 (55.1%) | 56879 (56.9%) | 23867 (55.8%) | 64349 (57.3%) | 25819 (55.4%) | 64077 (57.7%) | | 24705 (55.6%) |
| Private | 29970 (26.6%) | 13771 (29.0%) | 30659 (26.7%) | 13677 (28.8%) | 30651 (25.6%) | | 14028 (28.3%) | 25430 (25.4%) | 12043 (28.2%) | 28263 (25.2%) | 13037 (28.0%) | 27662 (24.9%) | | 12326 (27.7%) |
| Medicaid | 17412 (15.5%) | 6522 (13.7%) | 17209 (15.0%) | 6714 (14.1%) | 17262 (14.4%) | | 6452 (13.0%) | 14585 (14.6%) | 5504 (12.9%) | 16327 (14.5%) | 6151 (13.2%) | 15977 (14.4%) | | 5919 (13.3%) |
| Self-pay | 1103 (1.0%) | 616 (1.3%) | 1251 (1.1%) | 710 (1.5%) | 1227 (1.0%) | | 726 (1.5%) | 905 (0.9%) | 525 (1.2%) | 993 (0.9%) | 598 (1.3%) | 992 (0.9%) | | 578 (1.3%) |
| Other | 2616 (2.3%) | 1068 (2.2%) | 2355 (2.1%) | 948 (2.0%) | 2745 (2.3%) | | 1048 (2.1%) | 2150 (2.2%) | 832 (1.9%) | 2409 (2.1%) | 992 (2.1%) | 2323 (2.1%) | | 924 (2.1%) |
| **Income** |  |  |  |  |  | |  |  |  |  |  |  | |  |
| Highest income | 45042 (40.0%) | 21145 (44.5%) | 46142 (40.2%) | 21440 (45.1%) | 49434 (41.3%) | | 23099 (46.6%) | 42569 (42.6%) | 20089 (47.0%) | 49018 (43.6%) | 22471 (48.2%) | 49920 (45.0%) | | 21983 (49.5%) |
| Medium income | 31467 (28.0%) | 12716 (26.8%) | 32866 (28.6%) | 13019 (27.4%) | 35639 (29.8%) | | 14031 (28.3%) | 29410 (29.4%) | 12084 (28.3%) | 33318 (29.7%) | 13212 (28.4%) | 33459 (30.1%) | | 12738 (28.7%) |
| Lowest income | 34142 (30.3%) | 12873 (27.1%) | 34031 (29.6%) | 12339 (26.0%) | 32529 (27.2%) | | 11706 (23.6%) | 26378 (26.4%) | 9946 (23.3%) | 28423 (25.3%) | 10246 (22.0%) | 26028 (23.4%) | | 9092 (20.5%) |
| **Principal diagnosis category** |  |  |  |  |  | |  |  |  |  |  |  | |  |
| Acute MI | 15934 (14.2%) | 22042 (46.4%) | 15704 (13.7%) | 22115 (46.5%) | 15810 (13.2%) | | 22228 (44.9%) | 13130 (13.1%) | 19318 (45.2%) | 14708 (13.1%) | 20326 (43.6%) | 14809 (13.3%) | | 19461 (43.8%) |
| Angina | 870 (0.8%) | 83 (0.2%) | 849 (0.7%) | 79 (0.2%) | 912 (0.8%) | | 81 (0.2%) | 726 (0.7%) | 57 (0.1%) | 804 (0.7%) | 68 (0.1%) | 766 (0.7%) | | 67 (0.2%) |
| Ischemic Heart Disease | 35813 (31.8%) | 19183 (40.4%) | 36825 (32.1%) | 18994 (39.9%) | 37708 (31.5%) | | 20141 (40.6%) | 30066 (30.1%) | 16858 (39.4%) | 33880 (30.2%) | 18849 (40.5%) | 33839 (30.5%) | | 17718 (39.9%) |
| Other | 59886 (53.2%) | 6174 (13.0%) | 61484 (53.5%) | 6361 (13.4%) | 65147 (54.5%) | | 7108 (14.3%) | 56027 (56.1%) | 6538 (15.3%) | 62949 (56.0%) | 7354 (15.8%) | 61617 (55.5%) | | 7206 (16.2%) |
| **Facility Ownership** |  |  |  |  |  | |  |  |  |  |  |  | |  |
| Government | 6497 (5.8%) | 2840 (6.0%) | 6395 (5.6%) | 2604 (5.5%) | 7401 (6.2%) | | 2939 (5.9%) | 6397 (6.4%) | 2612 (6.1%) | 7000 (6.2%) | 2716 (5.8%) | 6536 (5.9%) | | 2500 (5.6%) |
| Investor | 16962 (15.1%) | 7381 (15.5%) | 17207 (15.0%) | 7243 (15.2%) | 18070 (15.1%) | | 7496 (15.1%) | 15052 (15.1%) | 6316 (14.8%) | 15782 (14.0%) | 6838 (14.7%) | 12996 (11.7%) | | 5666 (12.7%) |
| Missing | 3010 (2.7%) | 957 (2.0%) | 2950 (2.6%) | 1020 (2.1%) | 3277 (2.7%) | | 1179 (2.4%) | 2639 (2.6%) | 986 (2.3%) | 3255 (2.9%) | 1199 (2.6%) | 3262 (2.9%) | | 1277 (2.9%) |
| Non-Profit | 78521 (69.8%) | 34331 (72.3%) | 80635 (70.2%) | 34687 (73.0%) | 82560 (69.0%) | | 35755 (72.1%) | 68536 (68.6%) | 30732 (71.9%) | 77782 (69.2%) | 33599 (72.1%) | 79618 (71.7%) | | 33006 (74.3%) |
| University of California | 7513 (6.7%) | 1973 (4.2%) | 7675 (6.7%) | 1995 (4.2%) | 8269 (6.9%) | | 2189 (4.4%) | 7325 (7.3%) | 2125 (5.0%) | 8522 (7.6%) | 2245 (4.8%) | 8619 (7.8%) | | 2003 (4.5%) |

PCI: Percutaneous Coronary Intervention

SD: Standard deviation

MI: Myocardial infarction

**Appendix 3.** Utilization rate per 100,000 race-specific annual population


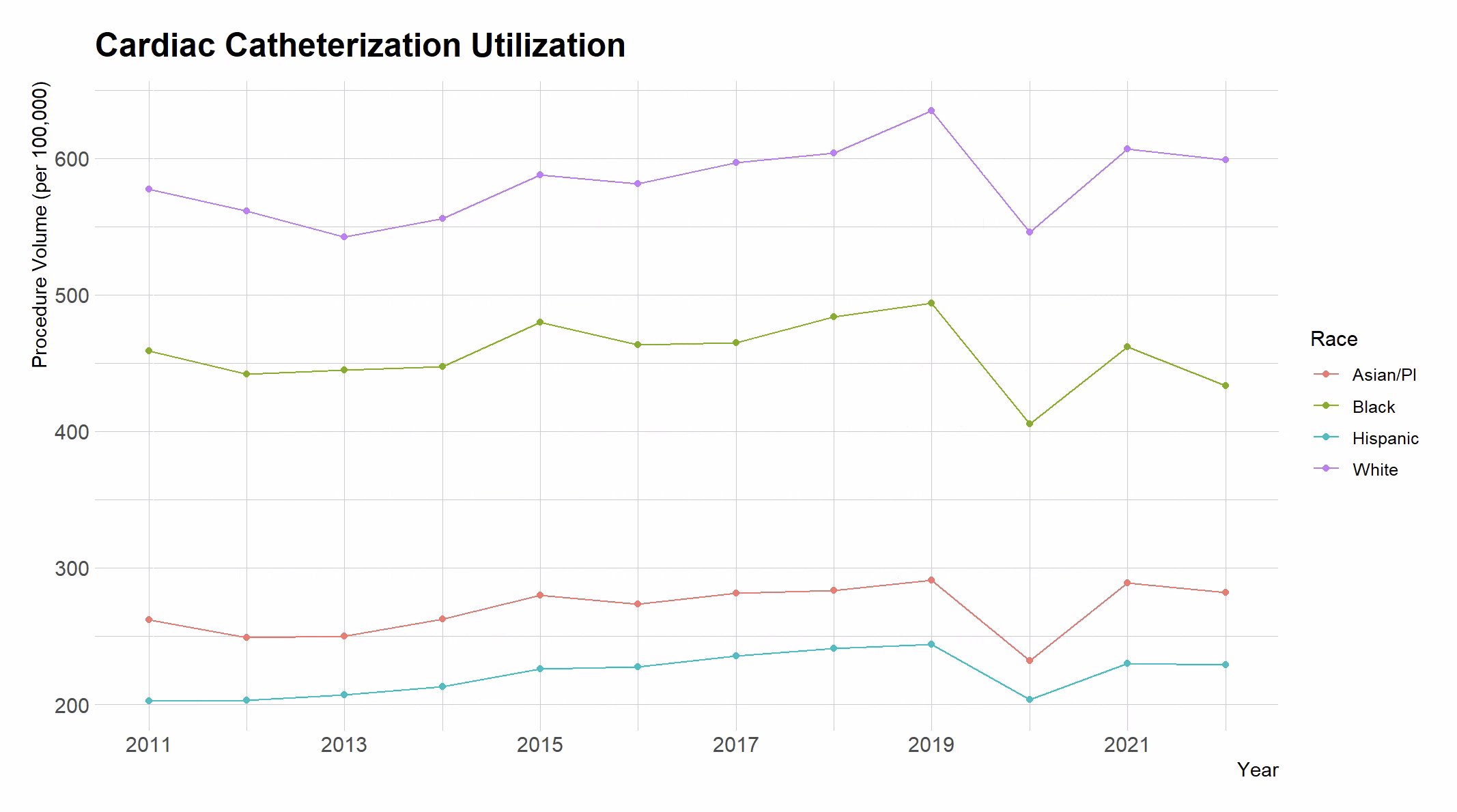


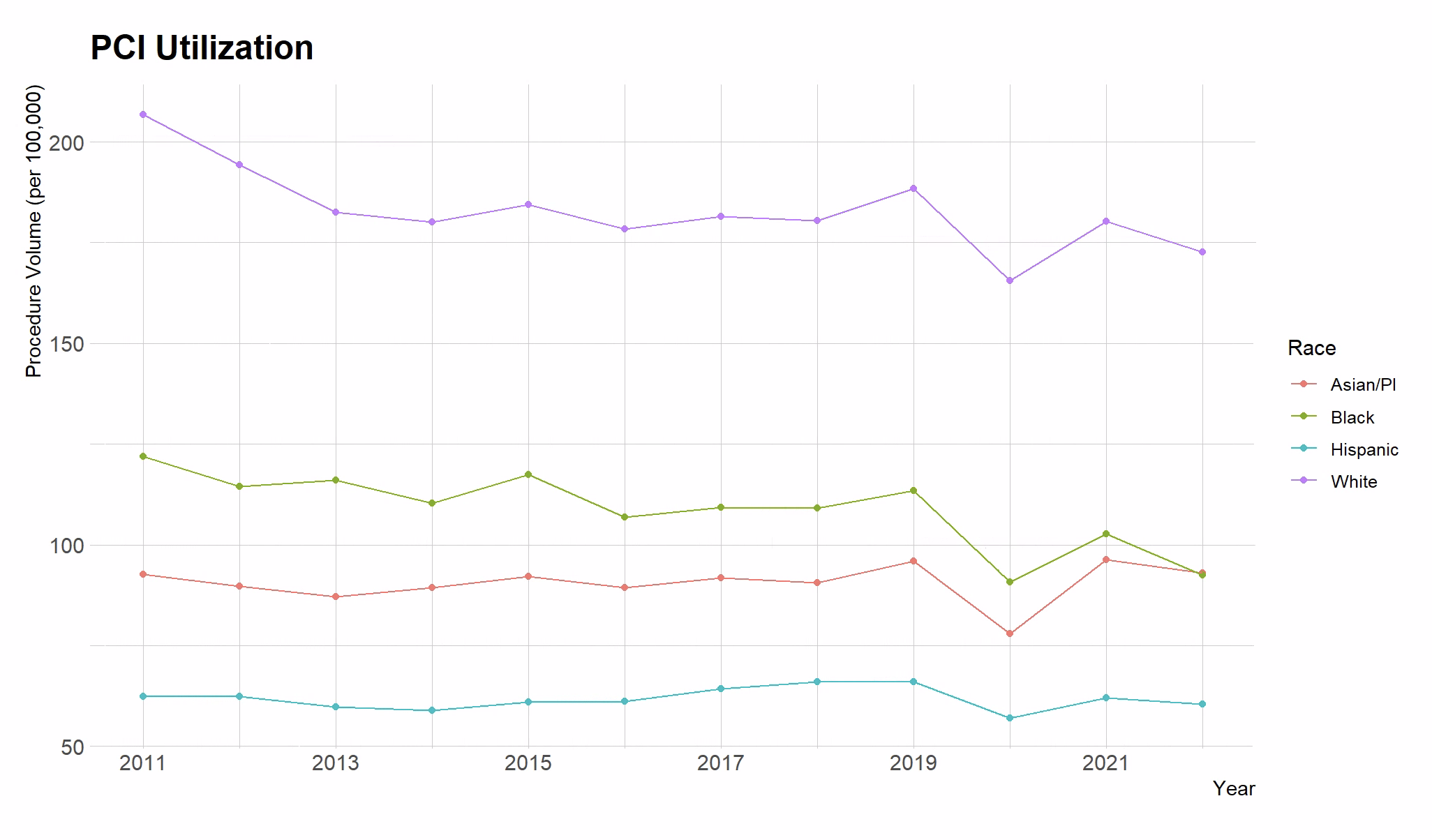


**Appendix 4.** Utilization by insurance type


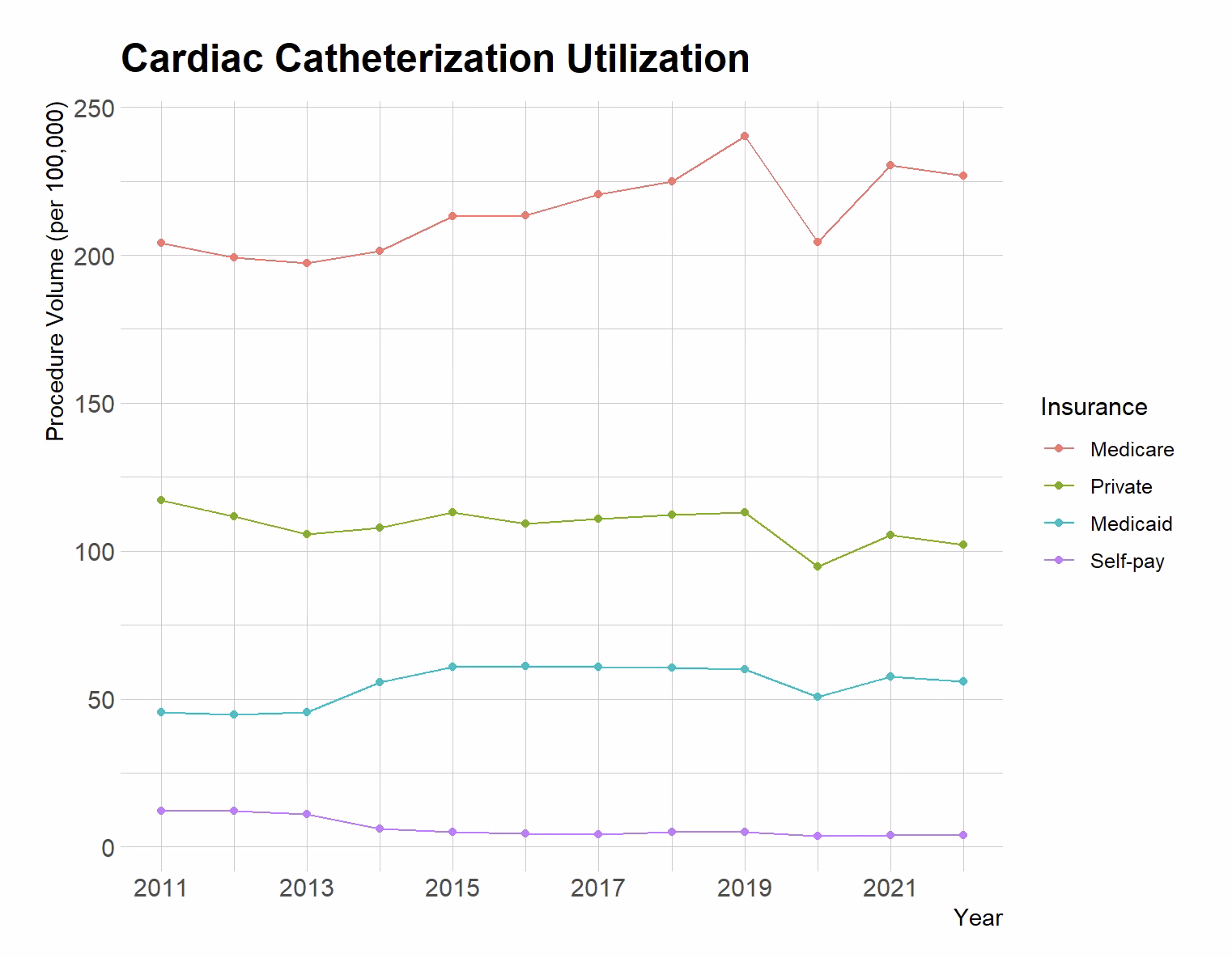


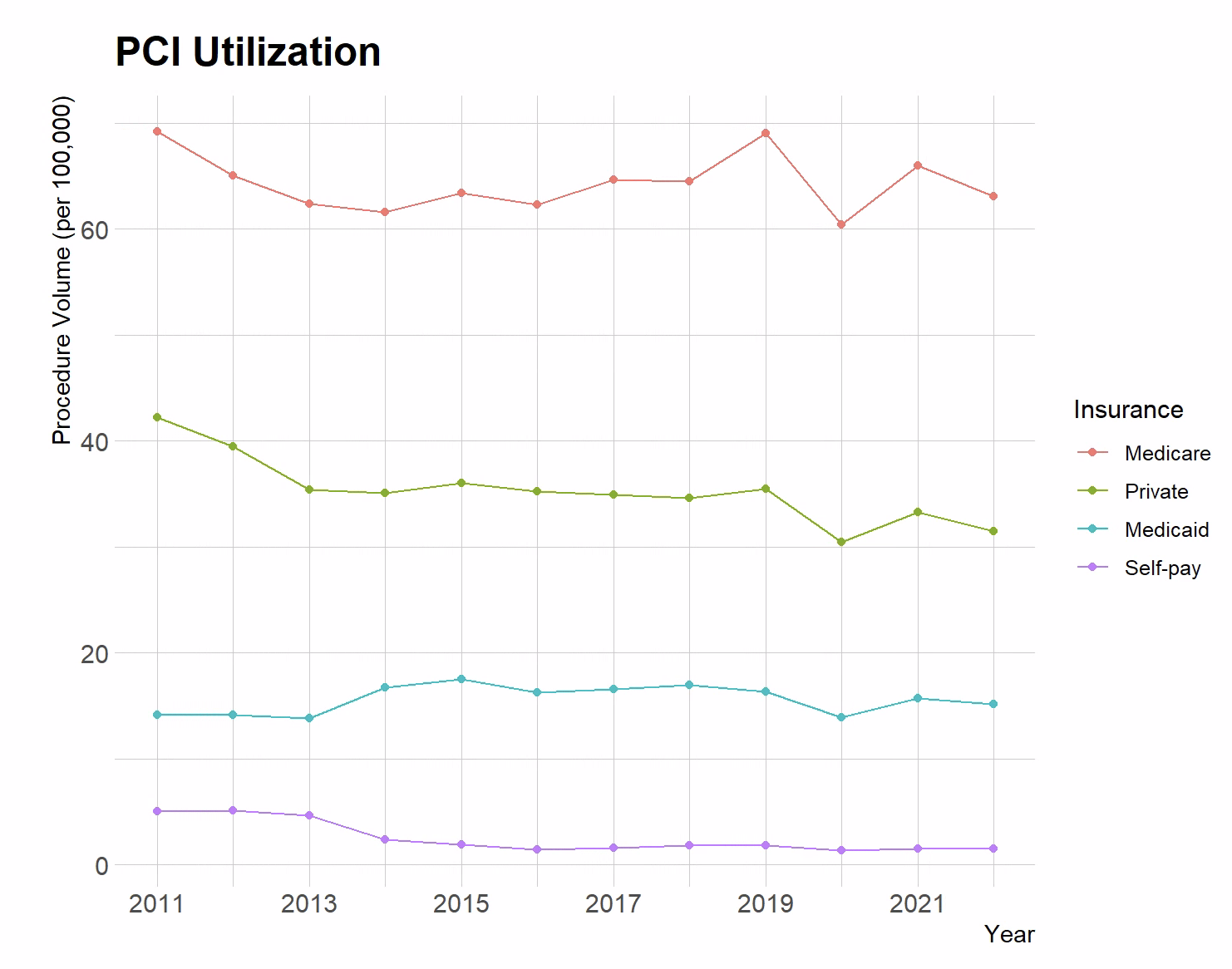


**Appendix 5.** Adjusted PCI share of catheterizations by race, payer, and income categories from a linear probability model adjusted for age, gender, race, payer, and income categories, with year fixed effects and cluster-robust standard errors

| **Race** | **Probability (%)** | **95% CI (%)** |
| --- | --- | --- |
| Asian/Pacific Islander | 33.1 | 32.4–33.9 |
| Black | 26.1 | 25.0–27.2 |
| Hispanic | 29.6 | 28.5–30.7 |
| White | 30.6 | 29.4–31.9 |
| Additional category | 34.8 | 33.7–35.8 |

Results are averaged over the distributions of age, gender, payer category, and income category

| **Payer** | **Probability (%)** | **95% CI (%)** |
| --- | --- | --- |
| Private | 32.1 | 30.9–33.3 |
| Public | 28.1 | 27.1–29.0 |
| Other | 32.4 | 31.3–33.5 |

Results are averaged over the distributions of age, gender, race, and income category

| **Income** | **Probability (%)** | **95% CI (%)** |
| --- | --- | --- |
| Highest income | 32.2 | 31.3–33.2 |
| Medium income | 30.5 | 29.5–31.5 |
| Lowest income | 29.8 | 28.8–30.9 |

Results are averaged over the distributions of age, gender, race, and payer category

CI: confidence intervals

**Appendix 6.** PCI share of cardiac catheterizations for Black and White patients, publicly or privately insured, by facility type

**
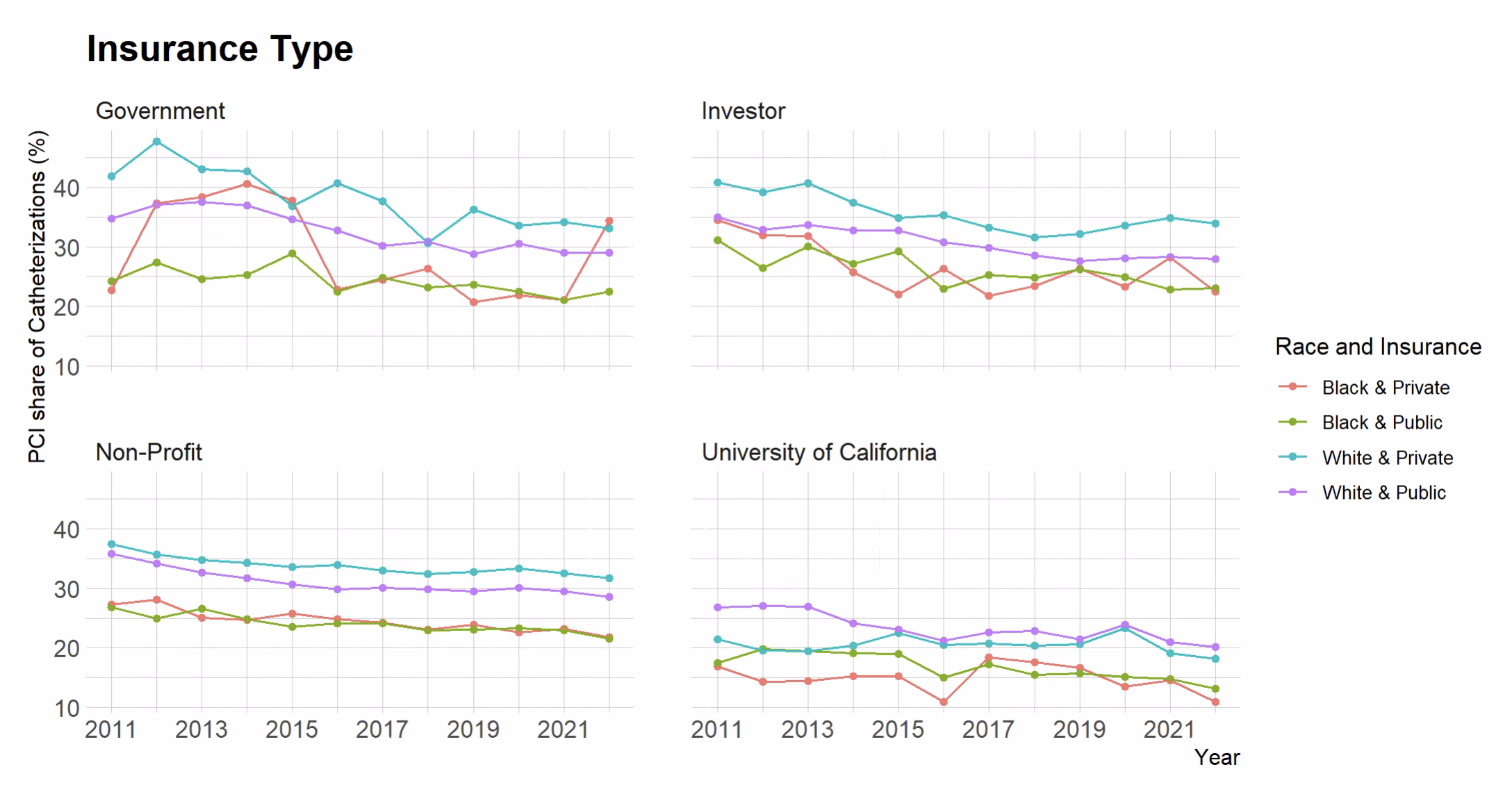
**

**Appendix 7: (A)** PCI share of cardiac catheterizations for patients with specific diagnosis categories of AMI, IHD, or STEMI; **(B)** revascularized share of cardiac catheterizations with either PCI or CABG within 1 day; and **(C)** total number of revascularized vessels as a share of cardiac catheterizations, assessed by household income category, public or private insurer, and Black or White racial category.

**A**


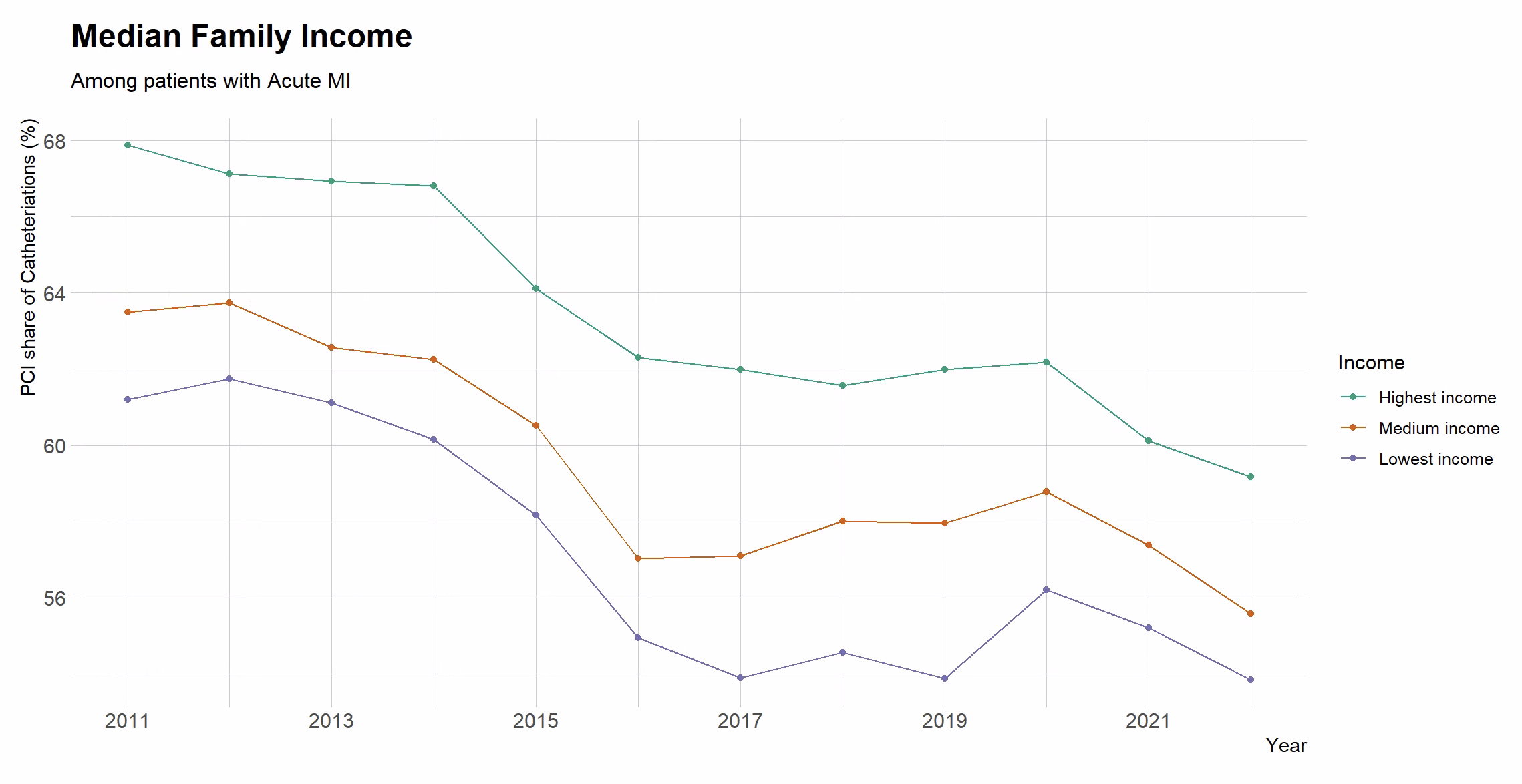


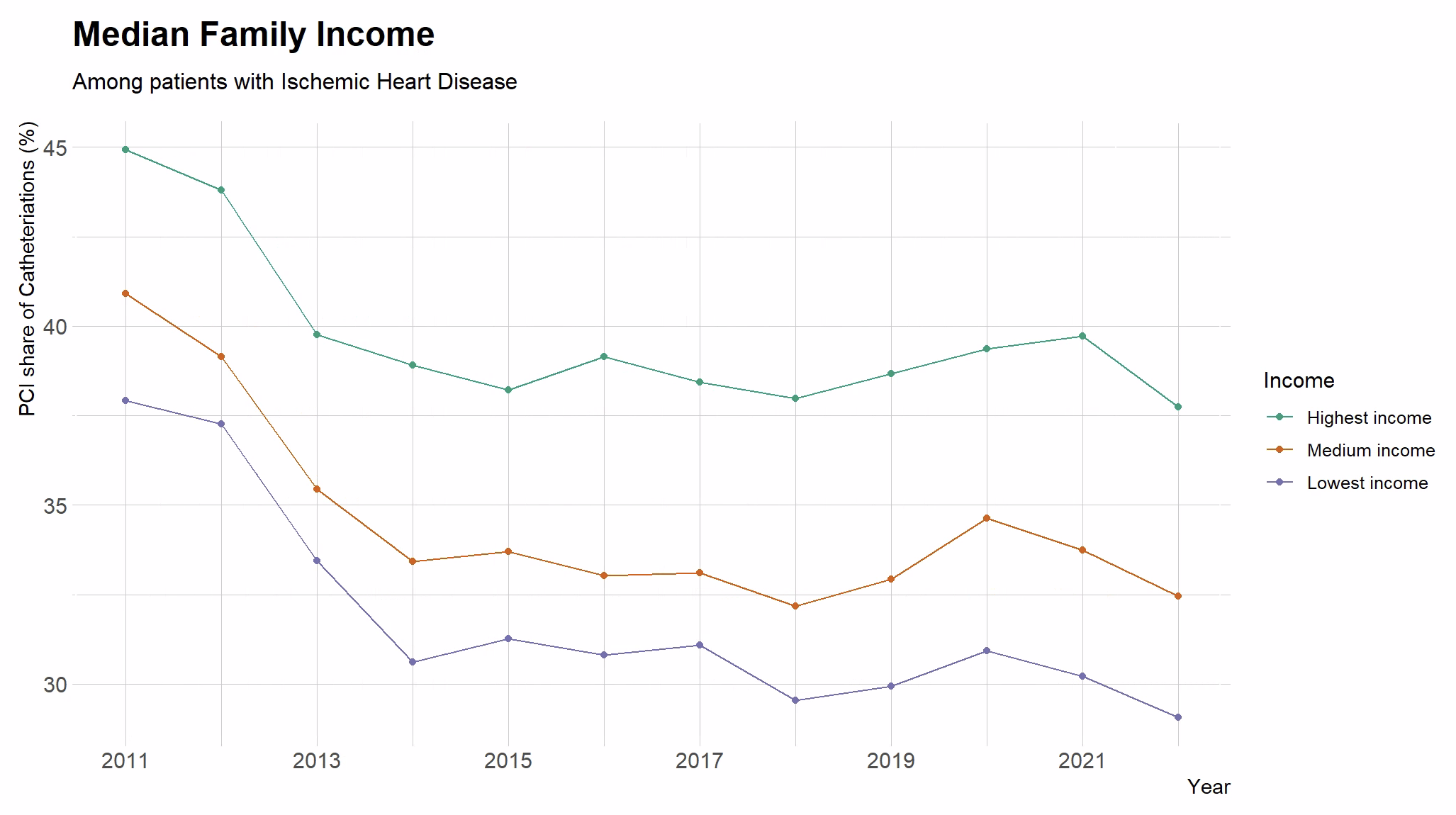


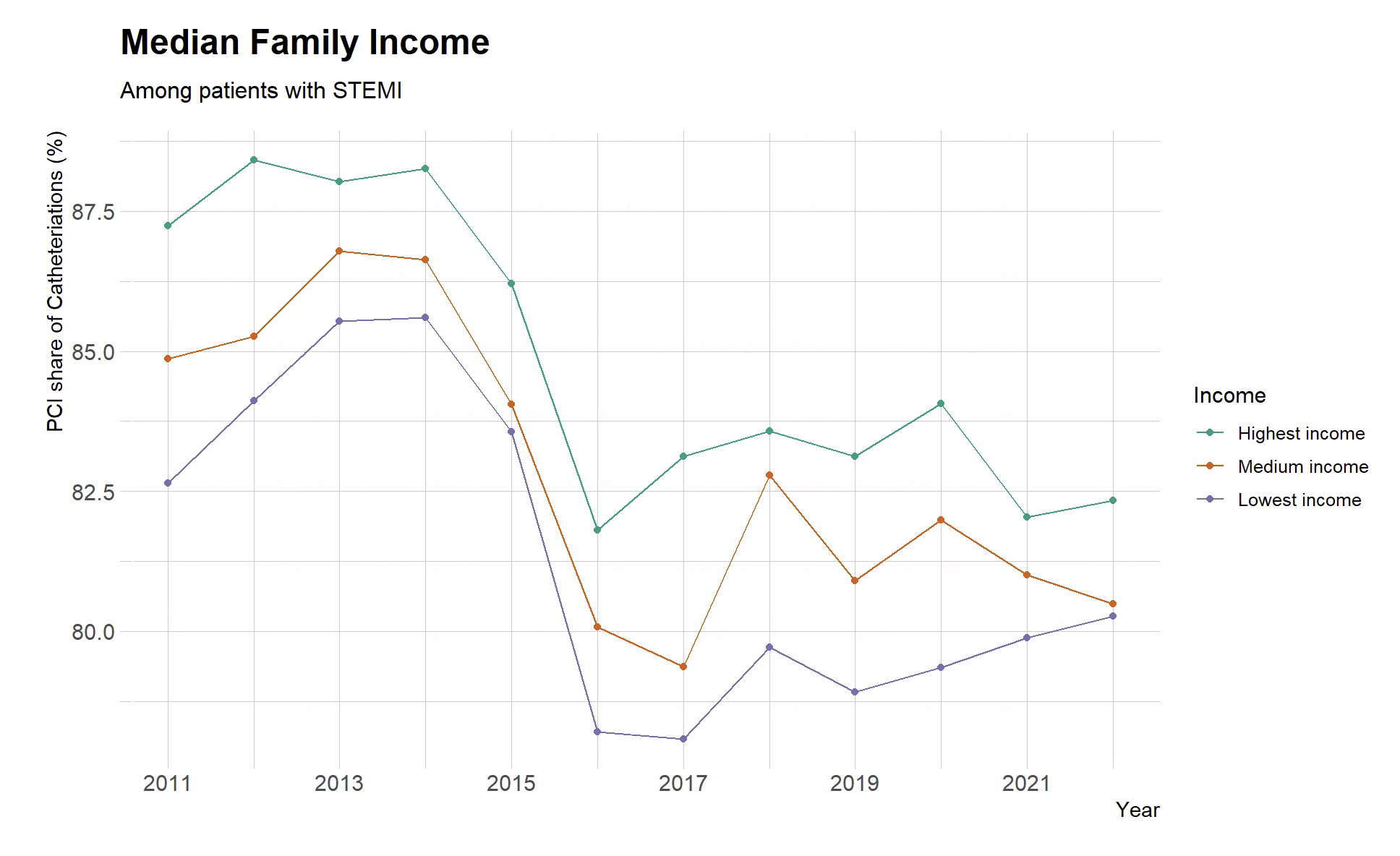


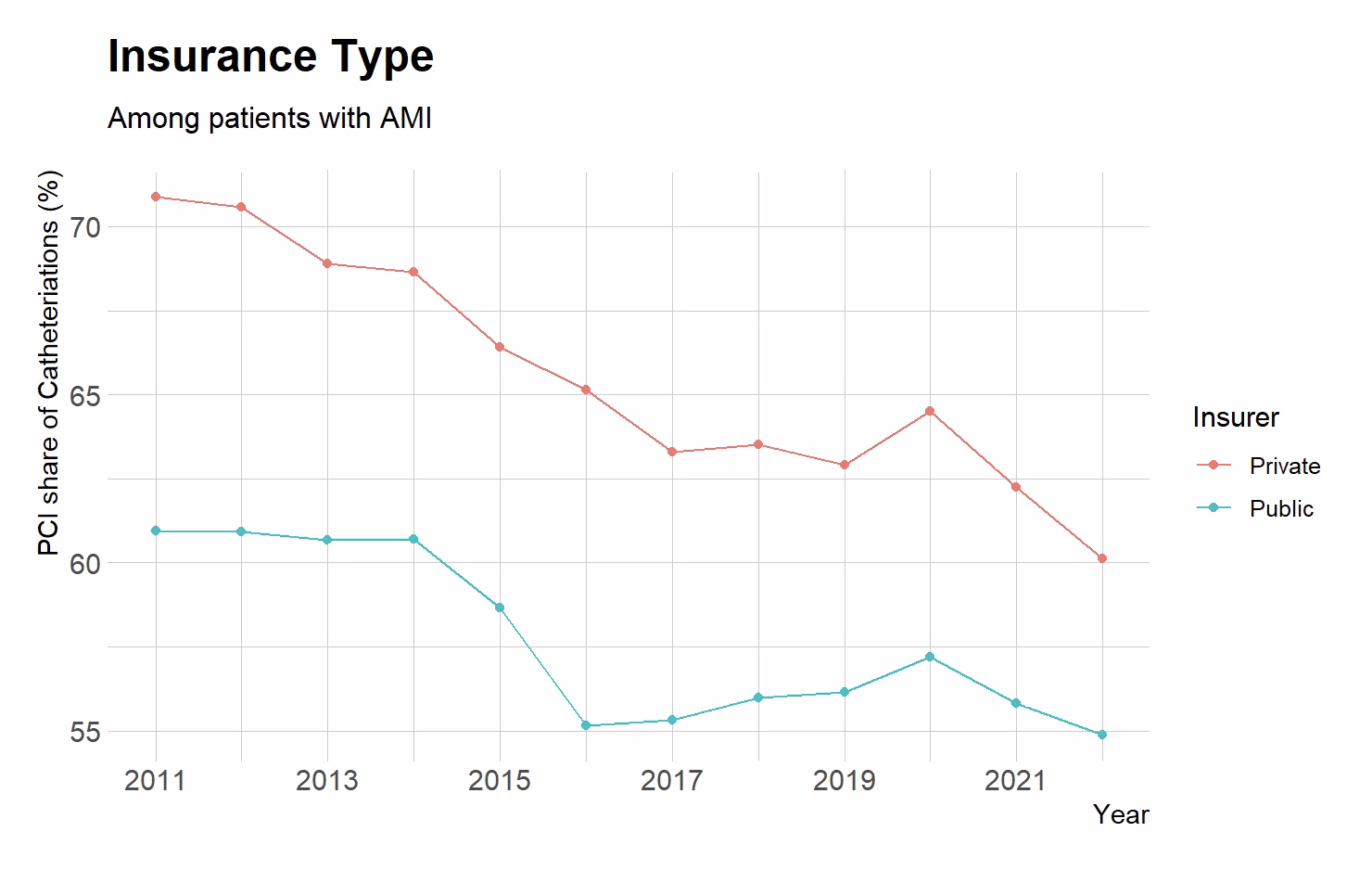


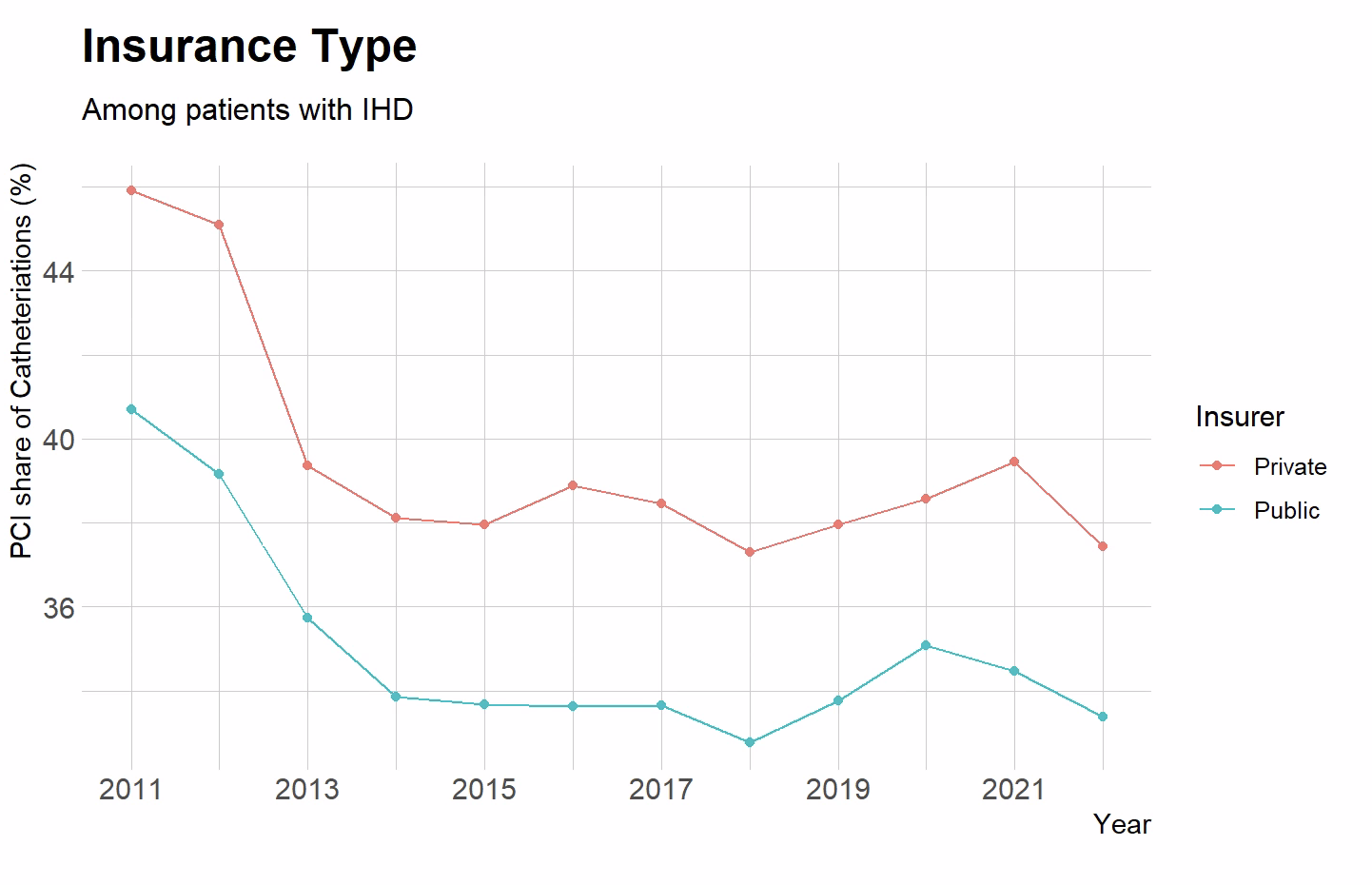


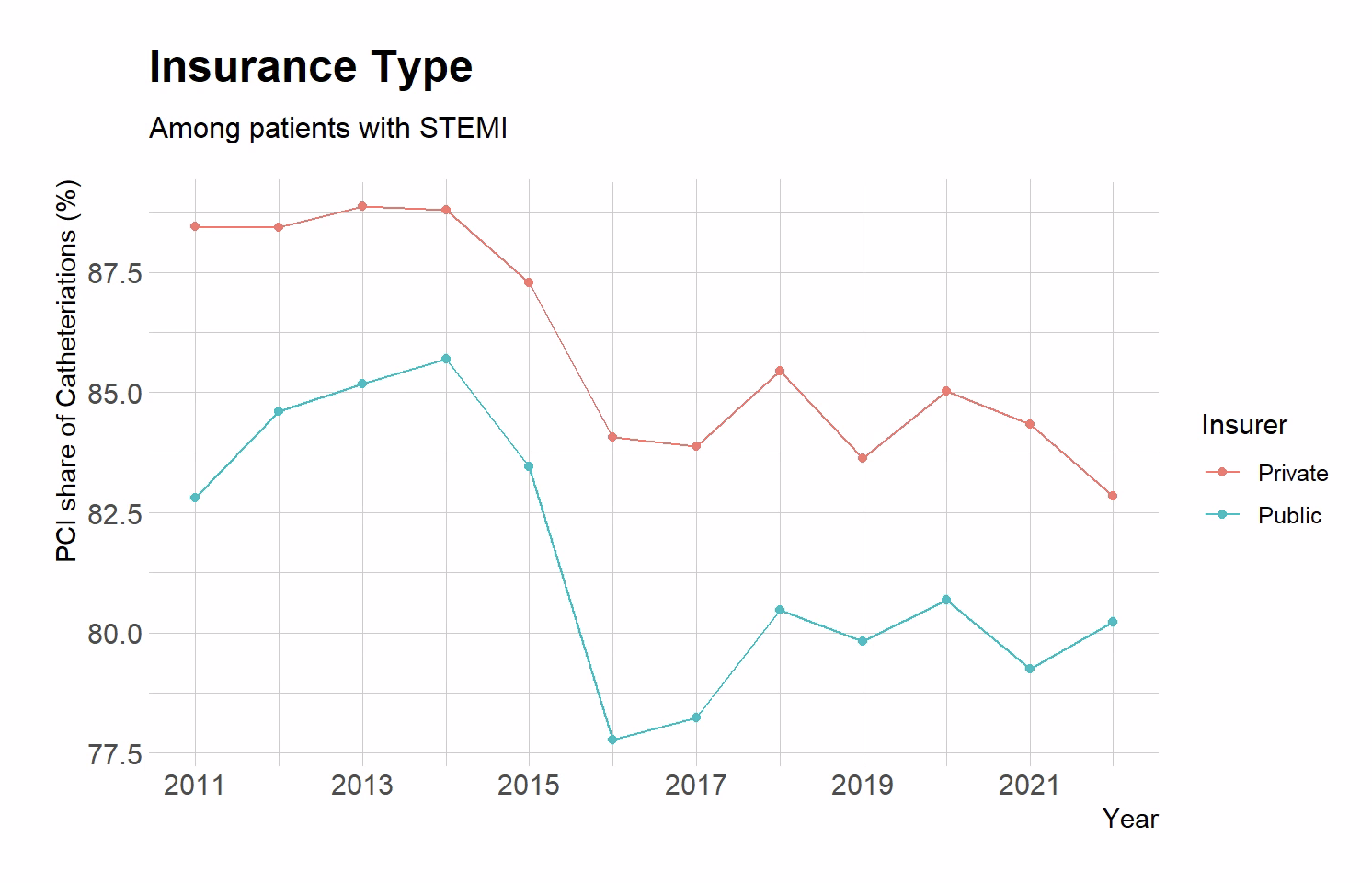


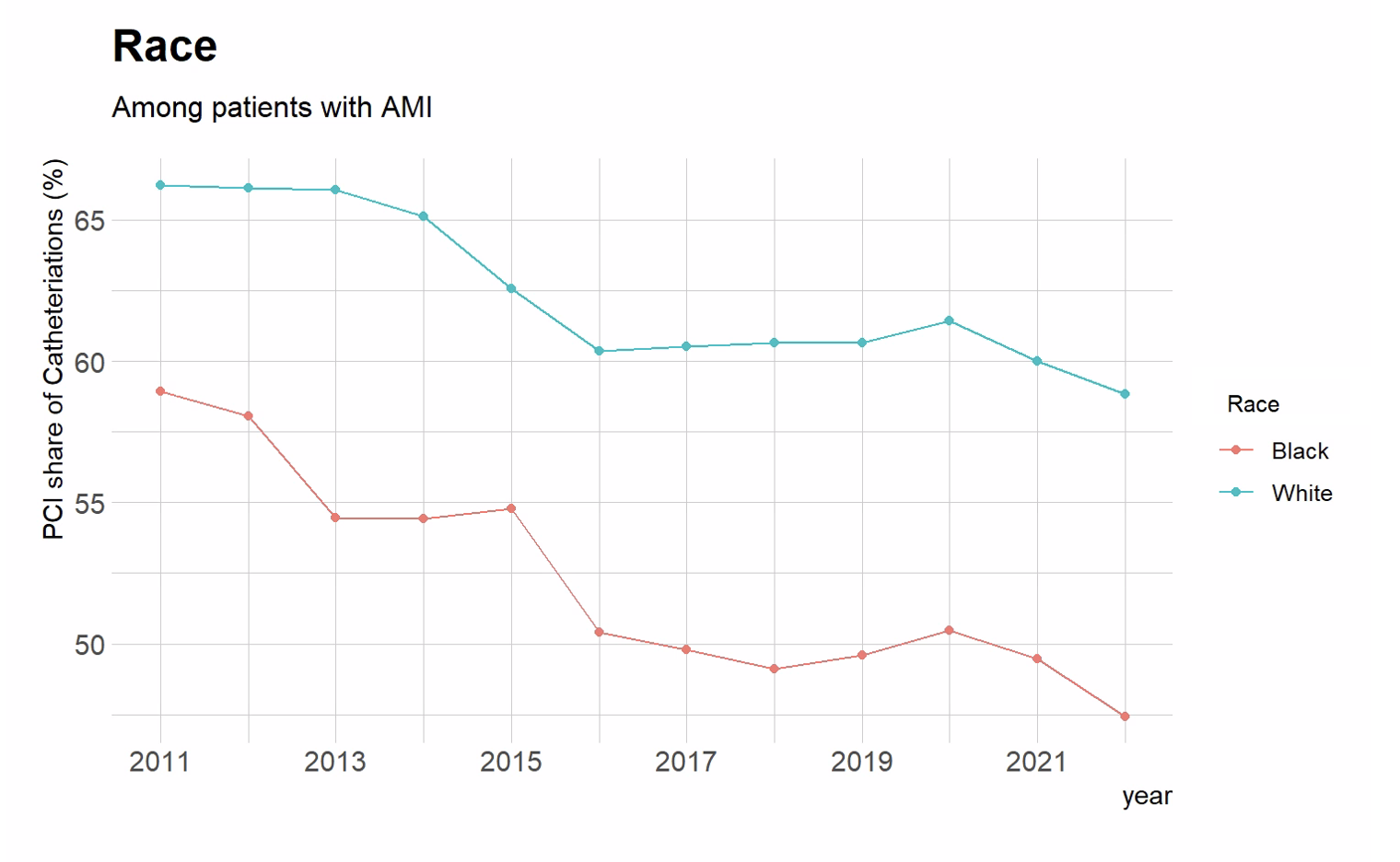


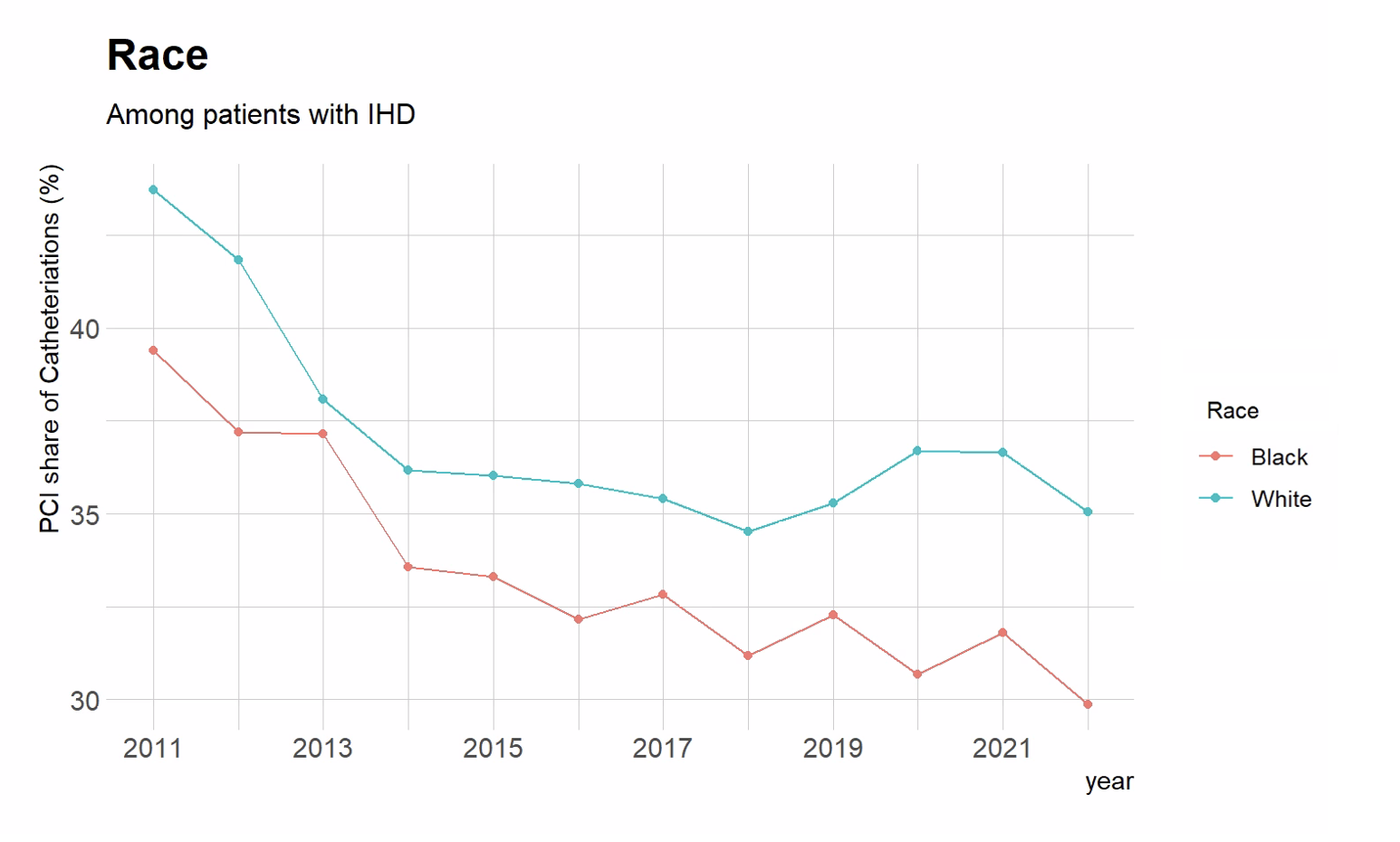


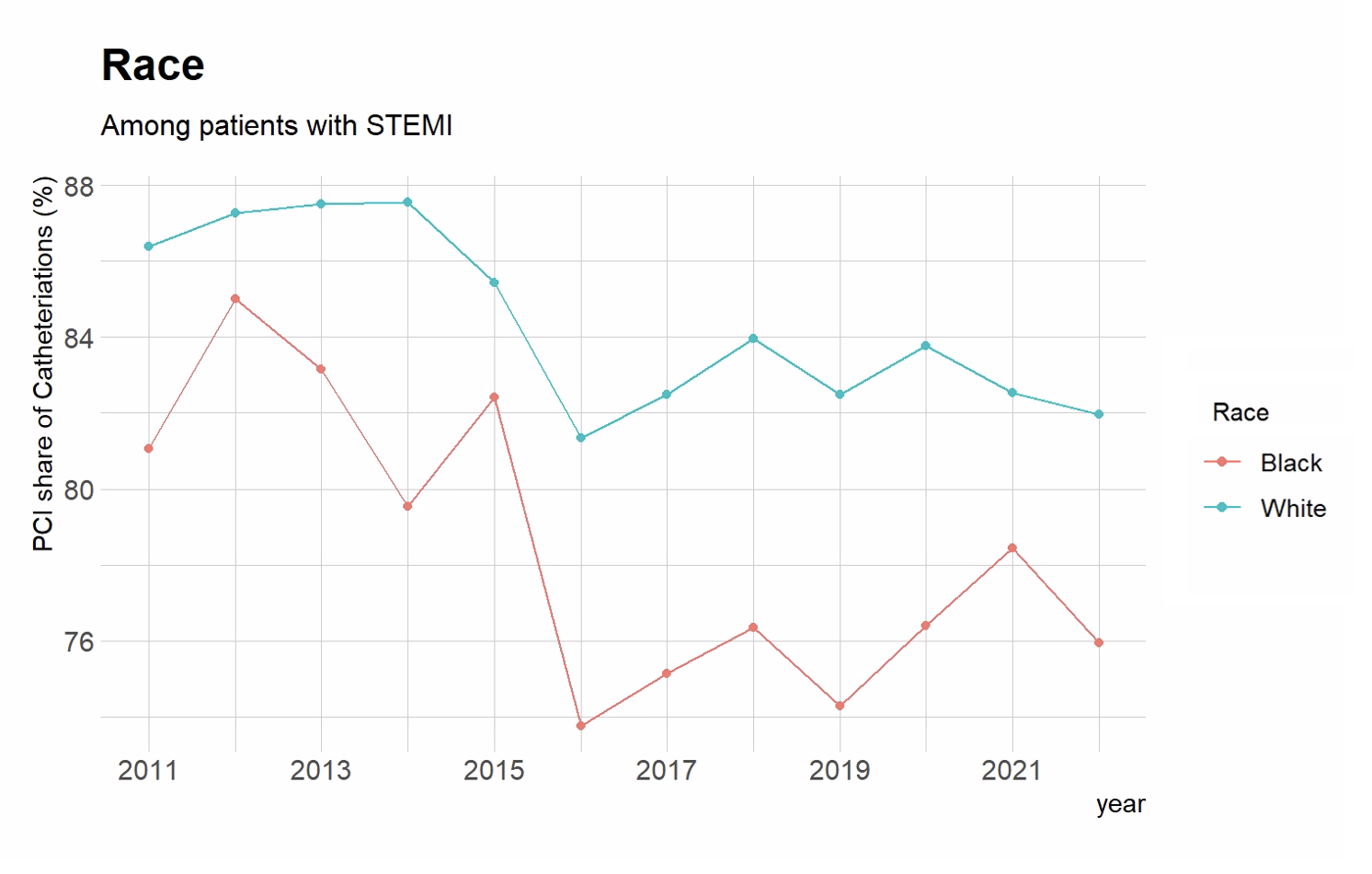


**B**


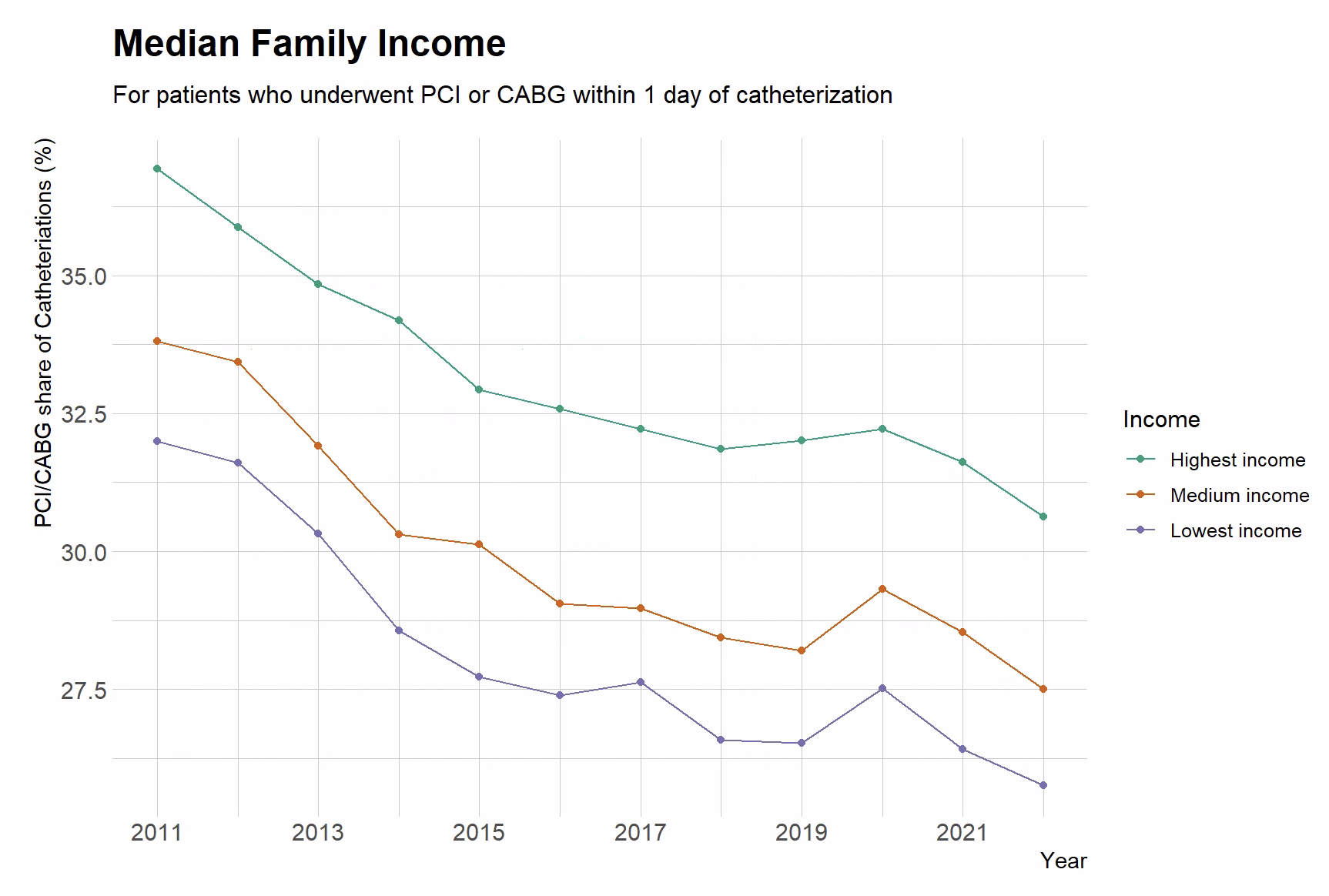


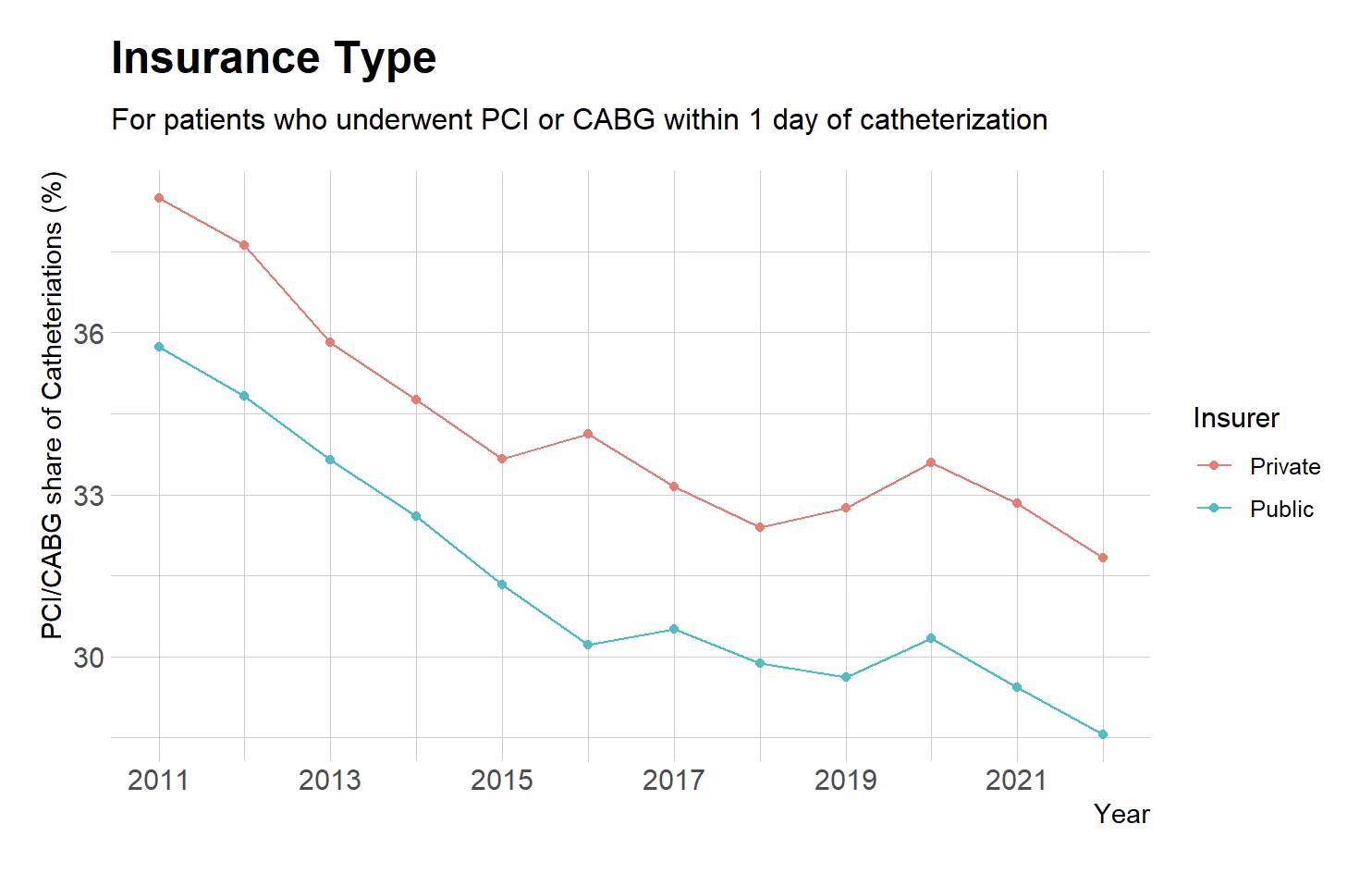


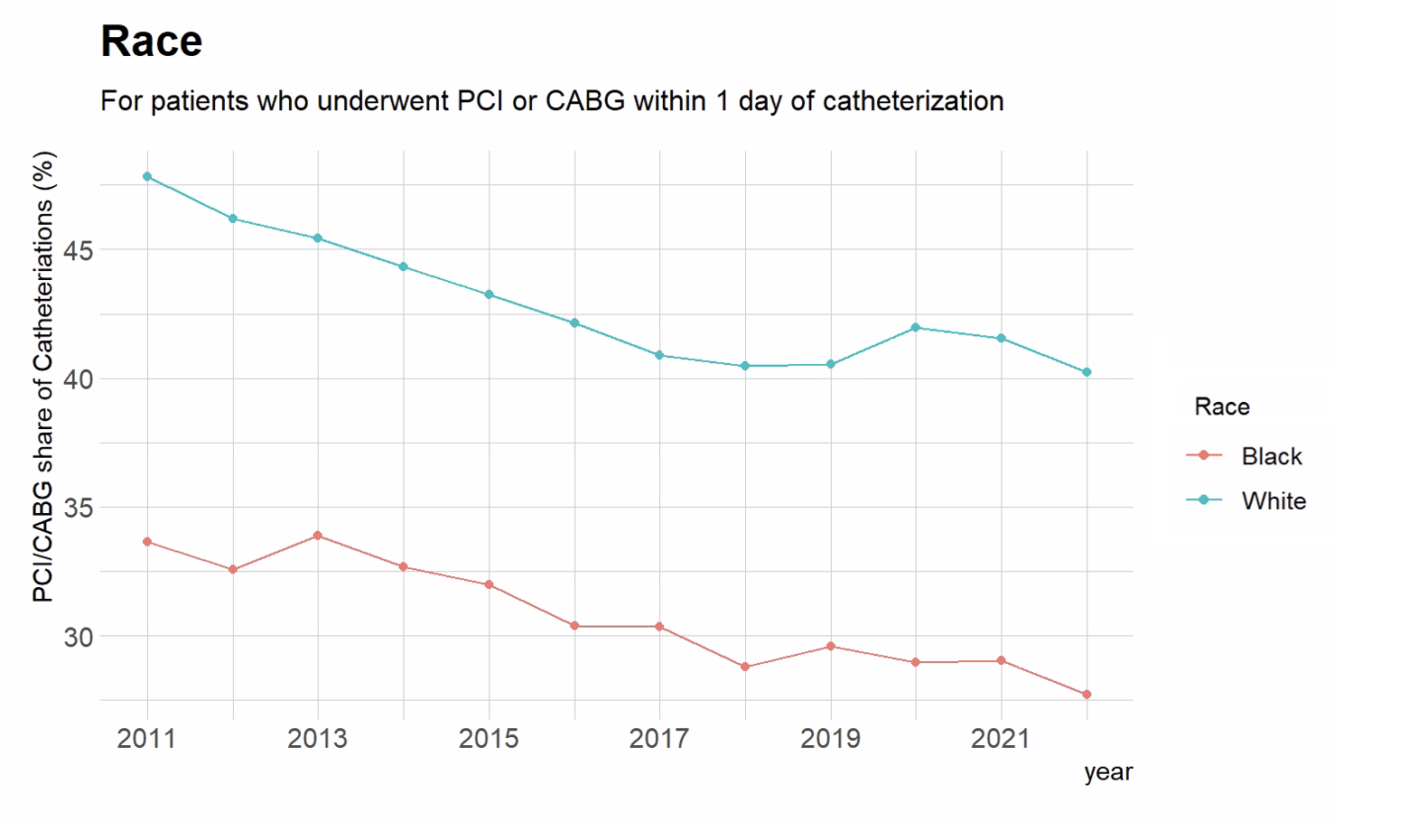


**C**


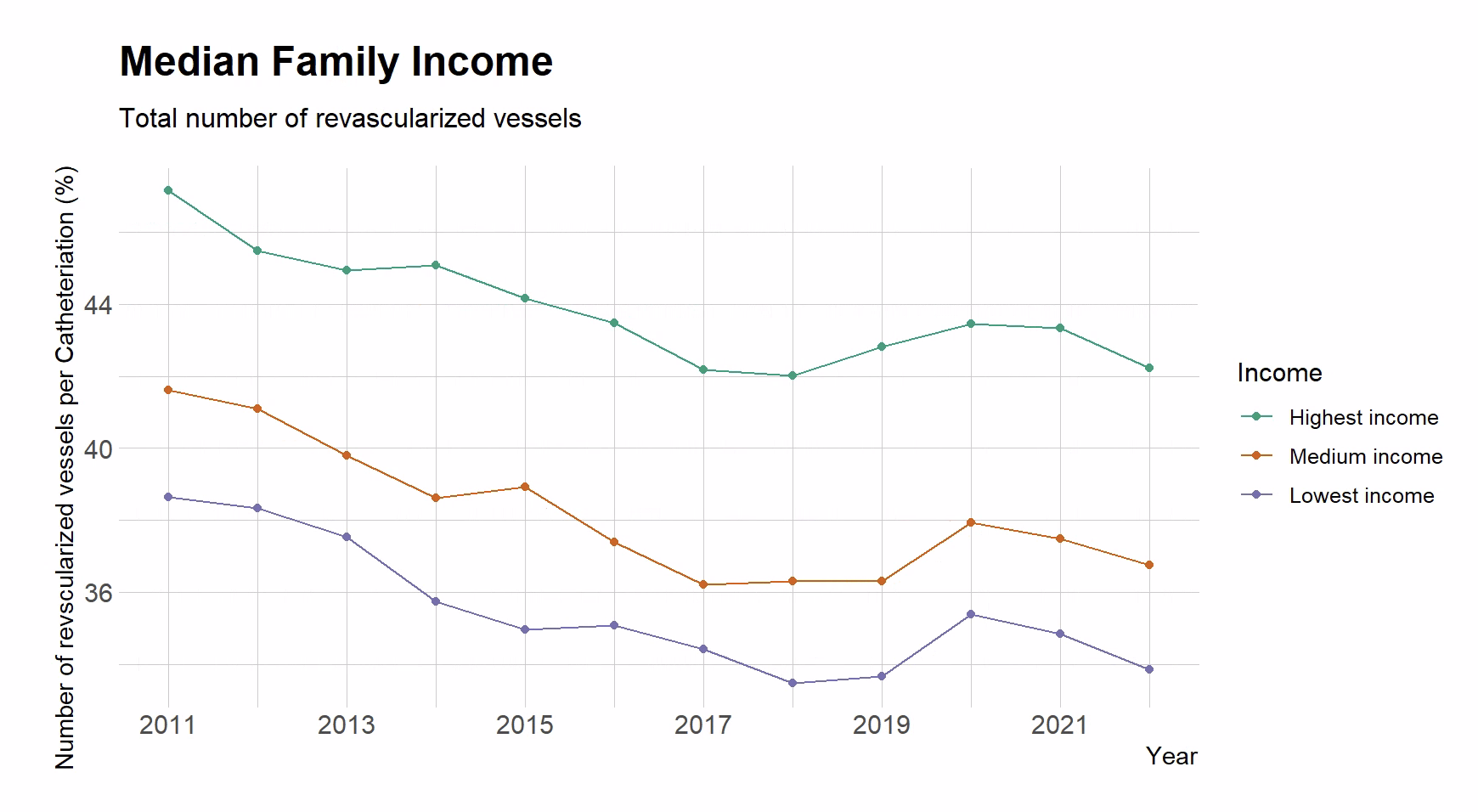


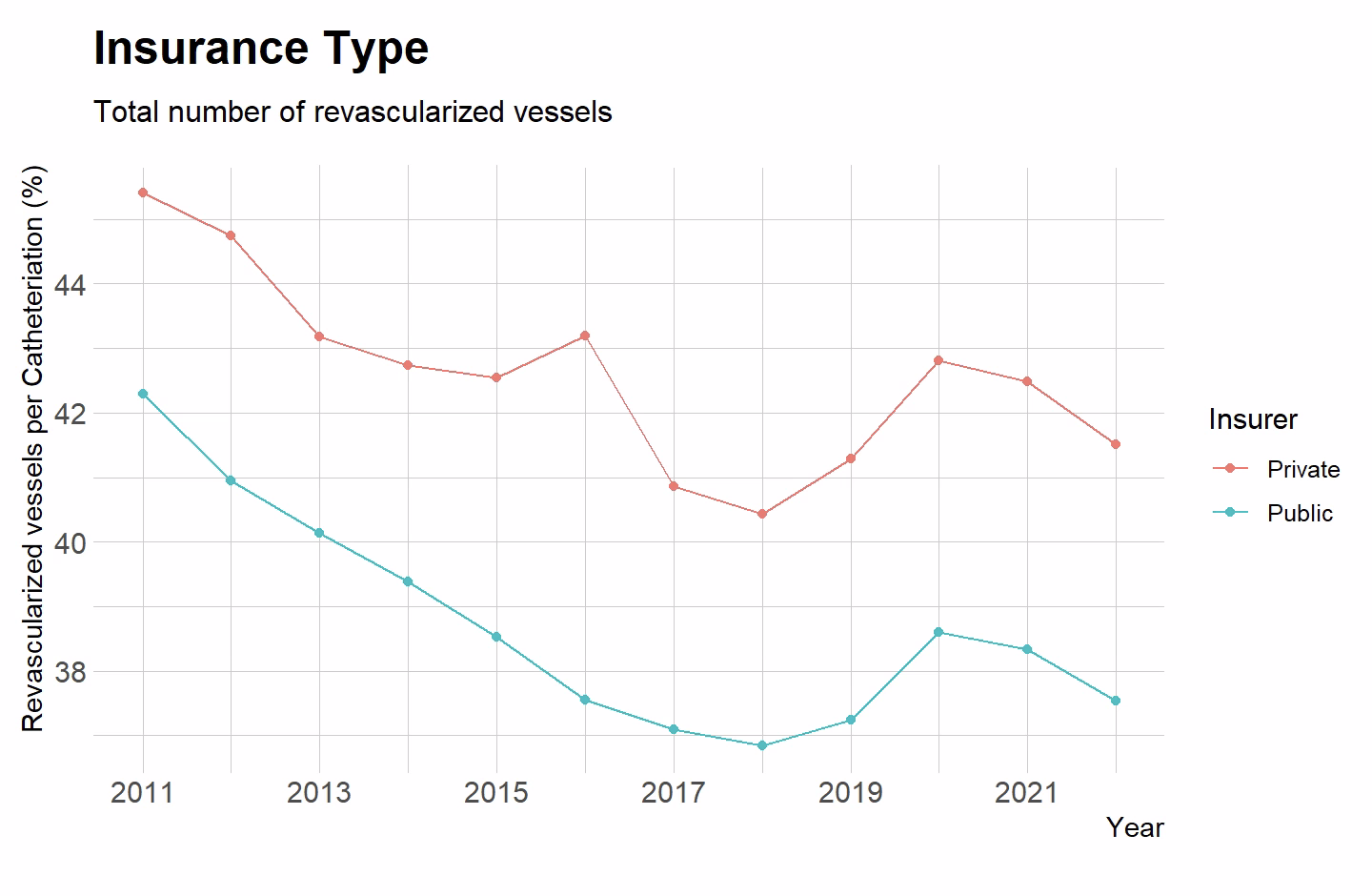


**
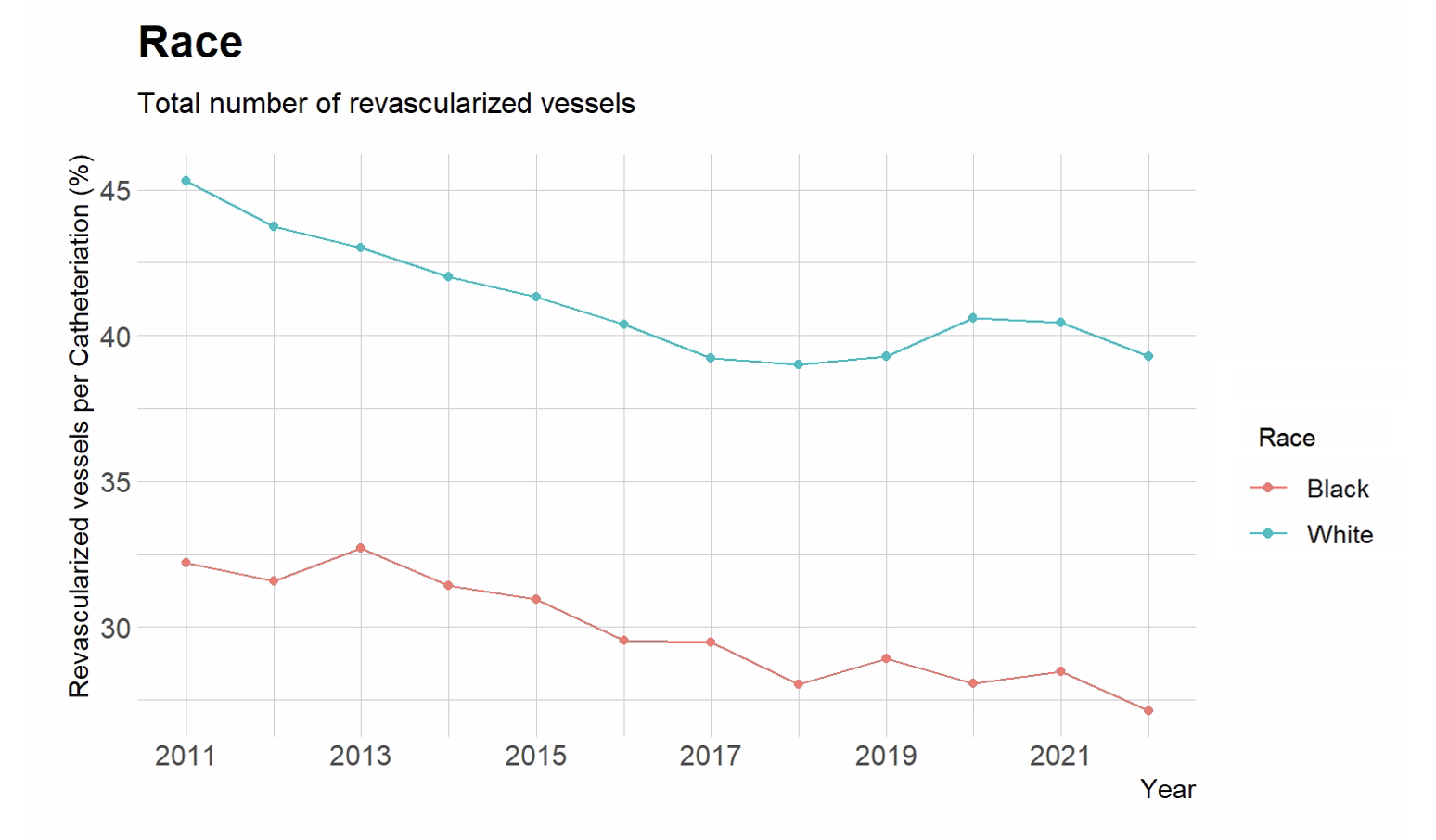
**

**Appendix 8.** Adjusted PCI share of catheterizations by race, payer, and income categories from a linear probability model adjusted for age, gender, race, year, payer, and income categories, with facility fixed effects and cluster-robust standard errors.

| **Race** | **Probability (%)** | **95% CI (%)** |
| --- | --- | --- |
| Asian/PI | 27.7 | 24.6–30.8 |
| Black | 20.5 | 17.4–23.7 |
| Hispanic | 24.4 | 21.4–27.4 |
| White | 25.4 | 22.4–28.4 |
| Additional category | 29.0 | 26.1–31.9 |

Results are averaged over the distributions of age, gender, year, payer category, and income category

| **Payer** | **Probability (%)** | **95% CI (%)** |
| --- | --- | --- |
| Private | 26.3 | 23.0–29.6 |
| Public | 22.6 | 19.4–25.8 |
| Other | 27.3 | 24.6–30.0 |

Results are averaged over the distributions of age, gender, race, year, and income category

| **Income** | **Probability (%)** | **95% CI (%)** |
| --- | --- | --- |
| Highest income | 25.4 | 22.3–28.5 |
| Medium income | 25.1 | 22.2–28.1 |
| Lowest income | 25.7 | 22.7–28.7 |

Results are averaged over the distributions of age, gender, race, year, and payer category

CI: confidence intervals
